# From Gelatin to GelMA: Versatility, Challenges, and Biomedical Applications of GelMA Hydrogels

**DOI:** 10.3390/gels12090844

**Published:** 2026-09-16

**Authors:** Federica Gemignani, Giulia Mesiano, Pompeo Marco Gaudiosi, Fabrizio Candido Pirri, Francesca Frascella

**Affiliations:** 1 Dipartimento di Scienza Applicata e Tecnologia, PolitoBIOMed Lab, Politecnico di Torino, 10129 Turin, Italy; federica.gemignani@hotmail.it (F.G.); pompeo.gaudiosi@polito.it (P.M.G.); fabrizio.pirri@polito.it (F.C.P.); francesca.frascella@polito.it (F.F.); 2 Center for Sustainable Futures, Istituto Italiano di Tecnologia, 10144 Turin, Italy

**Keywords:** gelatin methacryloyl (GelMA), hydrogels, biomaterials, reproducibility, standardization

## Abstract

Gelatin is a natural biopolymer derived from collagen and is widely employed in biomedical applications because of its biocompatibility, biodegradability, low toxicity, water solubility, and intrinsic thermo-responsive gelation behavior. Owing to the presence of bioactive motifs and its ability to form hydrogels under mild conditions, gelatin has emerged as a promising material for tissue engineering, drug delivery, and in vitro modeling. However, the poor mechanical stability and rapid dissolution of native gelatin under physiological conditions limit its direct use in advanced biomedical systems. To overcome these drawbacks, several chemical modification strategies have been developed, among which gelatin methacryloyl (GelMA) is one of the most investigated derivatives. GelMA combines the biological advantages of gelatin with photo-crosslinkable methacryloyl groups, enabling the fabrication of stable hydrogels with tunable mechanical, rheological, and degradation properties. This review adopts a source-to-performance perspective, systematically examining how gelatin origin, processing history, molecular characteristics, and Bloom strength may influence GelMA functionalization and subsequent hydrogel network formation. Particular attention is given to Type A and Type B gelatin, while recognizing that this classification does not fully capture the variability in the gelatin precursor. The review critically discusses how precursor characteristics interact with key synthesis and formulation parameters, including the degree of substitution/functionalization (DS/DoF), polymer concentration, photoinitiator content, and photo-crosslinking conditions, ultimately affecting network formation and the mechanical, rheological, swelling, porosity, degradation, and biological properties of GelMA hydrogels. This interconnected view highlights how variability introduced at the precursor level may propagate through functionalization and crosslinking, contributing to differences in GelMA performance and limiting direct comparison among reported formulations. The implications of these material-dependent properties are examined across three major biomedical application areas: tissue engineering, controlled drug delivery, and physiologically relevant three-dimensional in vitro models. Finally, current challenges and emerging opportunities related to GelMA standardization, biofabrication, multifunctional hydrogel design, personalized medicine, and clinical translation are considered. Overall, GelMA is presented not simply as a versatile biomaterial, but as a tunable protein-derived platform whose performance depends on the interconnected effects of precursor characteristics, functionalization, formulation, and network formation.

## 1. Introduction

Hydrogels have attracted considerable interest in biomedical research owing to their highly hydrated three-dimensional (3D) structures and their ability to mimic key features of the native extracellular matrix (ECM). Their high water content, often exceeding 90%, together with their porous architecture, allows them to closely resemble living tissues and provide suitable microenvironments for cell growth, proliferation, and tissue regeneration [1].

Among hydrogel-based biomaterials, gelatin methacryloyl (GelMA) has emerged as one of the most versatile platforms for a wide range of biomedical applications. Its relevance derives from the combination of the intrinsic biological properties of gelatin with the ability to form stable photo-crosslinked networks with tunable physicochemical and mechanical characteristics. Since its introduction, GelMA has been extensively investigated in tissue engineering, drug delivery, 3D cell culture, and biofabrication, and recent studies have further expanded its use toward advanced biomaterial inks, 3D/4D bioprinting, and physiologically relevant in vitro models [2,3,4,5].

The interest in GelMA should also be considered in the broader context of natural and synthetic hydrogel systems. Natural polymers such as collagen, hyaluronic acid, alginate, and gelatin provide intrinsic biological cues and generally support cell adhesion, proliferation, and tissue-specific interactions, but their mechanical properties, degradation behavior, and batch-to-batch consistency may be difficult to control depending on their source and processing history [6]. In contrast, synthetic polymers such as poly (ethylene glycol) (PEG) and its derivatives offer highly reproducible chemistry and extensive control over network architecture, mechanical properties, and degradation but are intrinsically bioinert and therefore often require the incorporation of cell-adhesive peptides, proteins, or other bioactive components to reproduce cell–matrix interactions [6]. GelMA occupies an intermediate position between these two classes by combining the biological functionality of a naturally derived protein with the covalent and spatially controlled crosslinking enabled by methacryloyl groups. In particular, GelMA retains cell-adhesive motifs and matrix-remodeling sites derived from gelatin while allowing the network properties to be modulated through the degree of functionalization, polymer concentration, and crosslinking conditions [7,8].

The distinctive feature of GelMA is therefore not that it universally outperforms other hydrogel systems, but that it provides a versatile platform in which biological functionality and network properties can be tuned within a single protein-derived material. This tunability has also enabled the development of hybrid GelMA systems that combine gelatin-derived bioactivity with the structural, mechanical, or functional advantages of other polymers [9]. Consequently, the performance of GelMA remains strongly dependent on both material composition and processing conditions, making the identification and control of the variables governing its properties particularly important.

Gelatin is obtained from collagen-rich animal sources, primarily porcine, bovine, and fish-derived tissues, through extraction processes that differ according to the pretreatment applied to the raw material. Collagen can undergo either acidic or alkaline pretreatment, leading to the production of Type A and Type B gelatin, respectively. Although Type A gelatin is commonly obtained from porcine skin and Type B gelatin is traditionally associated with bovine sources, the Type A/Type B classification primarily reflects the acidic or alkaline pretreatment used during gelatin production rather than the animal source itself [10]. These differences in source and extraction treatments influence the final composition of gelatin, including its amino acid, lipid, and carbohydrate contents, thereby affecting key physicochemical and functional parameters, such as thermo-reversibility, isoelectric point (IEP), and gel strength [11,12,13,14]. The increasing interest in alternative gelatin sources, including fish-derived materials, further highlights the importance of considering the origin and processing history of the precursor when evaluating gelatin-based biomaterials [15,16,17,18].

For biomedical applications, gelatin presents intrinsic limitations that prevent direct use in its native form, mainly due to its poor structural and mechanical stability, rapid degradation, and high solubility at physiological temperature. For these reasons, gelatin is often functionalized through physical, chemical, or enzymatic approaches to improve its performance and expand its applicability [19].

GelMA represents a relevant example of gelatin chemical functionalization. It is synthesized through the reaction between gelatin and methacrylic anhydride (MA), which introduces methacryloyl groups onto the amino and hydroxyl residues of the polymer backbone. Upon light exposure in the presence of an appropriate photoinitiator, these functional groups undergo covalent crosslinking, leading to the formation of the hydrogel [2,7,20]. Although GelMA is obtained through gelatin functionalization, it retains the bioactive motifs of the original polymer, including arginine–glycine–aspartic acid (RGD) sequences, which promote cell adhesion, as well as matrix metalloproteinase (MMP)-sensitive sites that enable cell-mediated degradation and matrix remodeling [21].

Importantly, the properties of the resulting GelMA are not determined exclusively by the methacrylation reaction itself but are also influenced by the characteristics of the gelatin precursors [22]. As observed for gelatin, several properties of GelMA are strongly linked to the nature of the precursor material. In this regard, Type A and Type B GelMA exhibit distinct physicochemical and mechanical behaviors, in terms of strength, viscosity, rheology, and sol-gel transition behavior. Importantly, however, the distinction between Type A and Type B does not fully capture the variability of the gelatin precursor. Characteristics such as gelatin source, molecular characteristics, and Bloom strength may also contribute to differences in the properties of the resulting GelMA hydrogels. For example, Aljaber et al. demonstrated that both gelatin source and Bloom number influenced the mechanical and biological properties of GelMA hydrogels, while Zambuto et al. subsequently showed that the Bloom strength of the starting gelatin affected the mechanical, viscoelastic, structural, and transport properties of the resulting GelMA networks [23,24].

Nevertheless, precursor characteristics represent only one component of the overall variability of GelMA. Synthesis and photo-crosslinking parameters, such as MA and photoinitiator concentrations, light intensity, and exposure time, also play a crucial role in defining the properties of the resulting hydrogel. Variations in these conditions directly influence GelMA chemical characteristics, affecting its degree of functionalization (DoF), degree of substitution (DS), and overall biocompatibility. Moreover, synthesis parameters regulate the organization of the hydrogel network, influencing pore size and matrix architecture, as well as the swelling capability and degradation rate of the hydrogel. Physical properties, such as hydrogel stiffness and rheological behavior, are also strongly dependent on the extent and density of network formation, ultimately determining the mechanical response of the material.

The importance of these parameters has recently prompted increasing attention toward GelMA standardization. He et al. proposed a framework in which the degree of substitution and solid content are considered key measurable parameters governing molecular network density and, consequently, GelMA porosity, viscosity, formability, mechanical strength, swelling, biodegradation, and cytocompatibility [21]. Similarly, recent work has emphasized the importance of controlling the rheological and crosslinking behavior of GelMA for advanced biofabrication. In particular, GelMA-based biomaterial inks require careful control of gelation, viscoelasticity, shear-thinning behavior, printability, and buildability, while the integration of such parameters with 3D/4D printing strategies is becoming increasingly relevant [3].

Together, these studies indicate that meaningful GelMA standardization requires consideration of both precursor characteristics and the parameters governing functionalization and network formation. These recent contributions illustrate that GelMA properties are governed by multiple interacting variables, ranging from precursor characteristics to methacrylation and subsequent network formation. Nevertheless, these variables are often investigated separately. Recent reviews have mainly focused on GelMA standardization and network-related parameters [14], multifunctional and nanomaterial-containing GelMA platforms [20], broad biomedical applications [2], or advanced fabrication and 3D/4D printing [3]. Although these studies have substantially advanced the field, the characteristics of the gelatin precursor are generally considered as one of several material variables rather than being systematically followed through the successive stages from gelatin production to GelMA functionalization, hydrogel network formation, and final biomedical performance.

**Figure 1:**
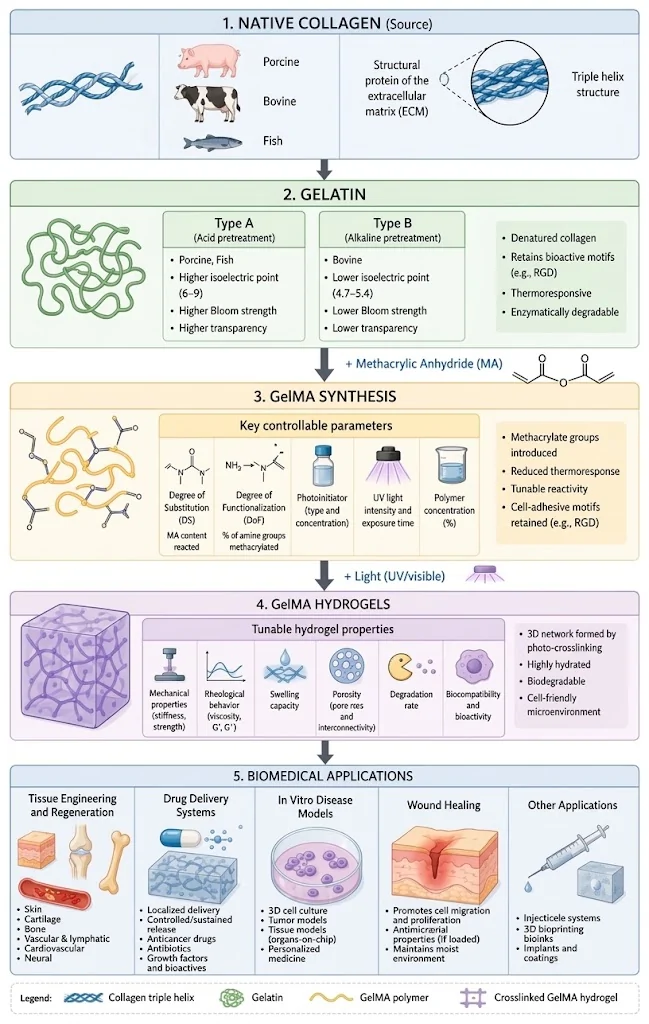
From collagen to GelMA: synthesis, key factors governing hydrogel properties, and representative biomedical applications.

A comprehensive source-to-performance analysis that explicitly connects these stages is therefore still lacking. The broad tunability arising from the interaction between precursor characteristics, functionalization, formulation, and crosslinking conditions enables GelMA to support diverse biomedical applications. In this review, these relationships are considered across three major application areas: tissue engineering, controlled drug delivery, and three-dimensional (3D) in vitro models, with representative examples selected according to how GelMA material properties influence biological and functional outcomes. An overview of the relationships among collagen, gelatin, and GelMA, together with the main factors governing GelMA properties and their impact on biomedical applications, is presented in Figure 1.

Accordingly, this review adopts a source-to-performance perspective, connecting collagen origin and gelatin processing with gelatin physicochemical characteristics, GelMA functionalization, hydrogel network formation, and final biomedical performance. Rather than providing another general overview of GelMA applications, we critically examine how variability introduced at the level of the gelatin precursor may propagate through subsequent synthesis and crosslinking steps and contribute to differences in the mechanical, rheological, swelling, porosity, degradation, and biological properties of GelMA-based hydrogels. Attention is dedicated to the differences between Type A and Type B gelatin and their influence on GelMA properties, while also considering the interaction between precursor characteristics and formulation parameters such as DS/DoF, polymer concentration, and photo-crosslinking conditions. Finally, the implications of these material-dependent properties are discussed across major biomedical applications, including tissue engineering, controlled drug delivery, and physiologically relevant three-dimensional in vitro models.

## 2. Gelatin: Fundamental, Sources and Properties

### 2.1. Collagen as the Precursor of Gelatin

Gelatin is a widely used and highly versatile biopolymer suitable for many biomedical applications not only due to its biodegradability, biocompatibility, water solubility, and low toxicity, but also because of its intrinsic ability to undergo thermally induced gelation, enabling the formation of physically crosslinked hydrogels at low temperatures. Gelatin is mainly composed of proteins, which represent between 85 and 92 per cent of the polymer, along with mineral salts, water, and small amounts of lipids [4,14]. It is derived from collagen, the primary component of the ECM in animal tissues and the most abundant structural protein in the human body. Collagen is classified into different molecular types according to its amino acid composition, molecular structure, chain organization, and tissue distribution [20,25]. This structural heterogeneity results in a tissue-specific distribution, which consequently determines the primary sources used for the extraction. For instance, Type I collagen is mostly derived from skin, bones, blood vessels, tendons, lungs, and cornea, whereas Type II originates from cartilage [26].

Collagen molecules undergo a hierarchical self-assembly process, first organizing into microfibrils with a diameter of approximately 40 nm. These microfibrils are further associated to form larger fibrils, typically ranging from 100 to 200 nm in diameter, which ultimately bundle together into collagen fibers with diameters between 50 and 500 nm. Each collagen molecule consists of a right-handed triple helix formed by three left-handed polypeptide α-chains, which are stabilized by covalent and hydrogen bonds, conferring rigidity and mechanical strength to the structure. Each α-chain consists of repeating glycine-Xaa-Yaa triplets, in which Xaa is predominantly proline and Yaa is principally hydroxyproline [19].

The structural organization and biochemical composition of the collagen precursor are relevant to gelatin production because the extraction and denaturation processes disrupt the native triple-helical architecture while preserving a heterogeneous population of collagen-derived polypeptide chains. Consequently, gelatin should not be considered a chemically uniform polymer, but rather a source- and process-dependent material whose molecular characteristics may subsequently influence its functionalization and the properties of the resulting GelMA hydrogel [23,27].

### 2.2. Sources of Gelatin and Extraction Processes

Gelatin is obtained from collagen-rich materials, primarily porcine skin, bovine hides and bones, and, to a lesser extent, fish collagen-containing materials such as fish skin, bones, scales, and muscles [4,14,15,28]. The extraction process, which mostly involves collagen Type I, consists of three main steps: pre-treatment of the raw material through acidic or alkaline processes, extraction of gelatin, and finally purification and/or drying of the product.

Extensive washing of the source of collagen is necessary to remove residual impurities and non-collagenous components, followed by a pre-treatment phase that depends on the origin of the material. Acidic pre-treatment, which is commonly applied to collagen derived from fish and porcine skin due to their lower degree of covalent crosslinking, induces primarily a physical rearrangement of the collagen network, with limited hydrolytic modification of the molecular structure. In contrast, alkaline pre-treatment is commonly used for bovine hides, which present a more extensively crosslinked structure. This process induces chemical modifications within the collagen matrix, promoting the disruption of the weaker interactions that maintain the native fibrillar architecture. Subsequent to this initial step, an acid or alkaline hydrolysis process is carried out, during which the triple helical structure of collagen is partially cleaved, leading to single-stranded polymeric molecules [4]. Acidic hydrolysis (at pH 1.0–2.0 [23]), conducted with the use of sulfuric or hydrochloric acid, leads to the formation of Type A gelatin, whereas alkaline hydrolysis (at pH 12.0–13.0) [23], performed under basic conditions, results in the production of Type B. The actual extraction of gelatin takes place by heating the material in hot water, after which it undergoes filtration and drying, and it is finally ground into a solid powder. Moreover, the degree of collagen conversion into gelatin can be optimized by adjusting hydrolytic processing parameters such as pH, temperature, and time [23,29].

Importantly, Type A and Type B gelatin are defined by the chemical pretreatment used during gelatin production rather than exclusively by the biological source. Although porcine and fish gelatin are commonly obtained through acidic processing and bovine gelatin is frequently produced through alkaline treatment, the relationship between gelatin type and animal source is not absolute. Therefore, the gelatin source and extraction process should be considered as related but distinct variables when comparing gelatin-based materials.

This distinction is particularly relevant for GelMA because the pretreatment history of gelatin contributes to differences in molecular characteristics, charge distribution, and functional group availability, which may subsequently affect methacrylation and the properties of the resulting hydrogel. Experimental studies have indeed demonstrated that GelMA derived from gelatin of different biological sources can exhibit markedly different mechanical and biological properties [23].

The extraction conditions, together with pre-treatment methods and the source of collagen, significantly affect both gelatin chemical composition and physical properties. For instance, gelatin derived from older animals usually provides higher extraction yields and tends to exhibit increased levels of fat and ash. Conversely, prolonged or high-temperature extraction is associated with reduced viscosity and diminished gel-forming capacity [30,31]. Thus, variability may already be introduced at the gelatin production stage, before any chemical functionalization is performed. This upstream variability represents an important consideration when comparing GelMA materials prepared from nominally similar gelatin sources.

### 2.3. Chemical Composition and Molecular Structure

Chemically, gelatin can be described as a polydisperse protein consisting of a complex and heterogeneous mixture of polypeptide chains. Its molecular weight distribution reflects the coexistence of distinct structural components, mainly α-chains and their covalently associated oligomers. Gelatin is primarily composed of free α-chains, β-chains (dimers of α-chains covalently crosslinked), and γ-chains (trimers of α-chains covalently crosslinked), with approximate molar masses of 90 × 10^3^, 180 × 10^3^, and 300 × 10^3^ g/mol, respectively [30,31].

Similar to collagen molecules, gelatin is made of α-chains mostly characterized by the repetitive glycine–proline–hydroxyproline triplet motif, which is fundamental in the formation of triple collagen-like helices. In particular, hydroxyproline is considered to have a unique function in stabilizing the triple-stranded collagen helix due to its ability to form hydrogen bonds through its -OH group [27,32]. Gelatin amino acid composition is therefore dominated by glycine (~21%), proline (~12%), and hydroxyproline (~12%), along with other residues such as arginine, alanine, aspartic acid, leucine, serine, lysine, and valine, all covalently linked through peptide bonds [4].

The content of proline and hydroxyproline in gelatin is closely related to its origin; in the case of fish-derived gelatin, the levels of these amino acids are generally lower compared to mammalian gelatin, especially in cold-water fishes such as cod, hake, and Alaska pollock. For gelatin obtained from warm-water fishes such as tilapia, tuna, and black carp, instead, the amino acid content is more similar to those found in porcine and bovine gelatins [14,17,28]. These compositional differences contribute to the distinct thermal and gelation behavior of fish gelatin and should be considered when evaluating its suitability as a precursor for GelMA. Fish gelatin has attracted increasing interest as an alternative gelatin source because of its distinct physicochemical characteristics and potential to overcome some limitations associated with mammalian-derived materials [14,17,28].

In addition to the amino acid composition, there are other significant differences between fish and mammalian gelatin. One of these is related to the lipid content, particularly the polyunsaturated fatty acids, such as omega-3 fatty acids, found in fish skin. Fish skin is also characterized by the presence of bioactive peptides, including those with anti-cancer, antioxidant, anti-inflammatory, immune-regulatory, anti-atherosclerotic, antihypertensive, and antimicrobial properties, may contribute to its biological activity [14,17,28].

Another notable distinction between fish and mammalian gelatin is the presence of hydroxyapatite in fish scales [17]. However, these characteristics are strongly dependent on the specific fish species and tissue source and should therefore not be generalized to all fish-derived gelatin.

The source of gelatin and the analytical methods employed to obtain it also influence the content and the specific type of sugars present in the gelatin itself. Chemical composition analyses have shown that gelatin contains approximately 1% of carbohydrates. Commonly identified sugars include galactose, glucose, mannose, lactose, and xylose, which may occur in the form of amino sugars. These carbohydrates are generally attributed to the presence of mucopolysaccharides associated with the collagen matrix, which act as structural binding components, contributing to gelation processes and influencing the chemical behavior of gelatin [18].

Gelatin is also characterized by the absence or minimal content of aromatic amino acids (i.e., tyrosine, tryptophan, and phenylalanine), resulting in favorable biocompatibility, which is further enhanced by the presence of biologically active motifs, such as the RGD sequence, that support integrin-dependent cell attachment [4,31].

Overall, gelatin composition is determined by a combination of biological source, tissue of origin, animal age, extraction conditions, and subsequent processing. This compositional heterogeneity is particularly relevant for GelMA because amino-containing residues, including lysine and hydroxylysine, provide important sites for methacrylation. Consequently, differences in the abundance and accessibility of these functional groups may affect the extent of gelatin modification and ultimately contribute to differences in GelMA network formation and hydrogel properties [23].

### 2.4. Physicochemical Properties of Gelatin

#### 2.4.1. Thermo-Reversible Gelation

Within gelatin chains, non-polar domains, arising from the repetition of glycine-proline-hydroxyproline sequences, alternate with polar regions: the distribution of these amino acid functionalities influences the physicochemical properties, including melting and gelling temperature, of the final material. The presence of polar and charged residues enhances gelatin’s ability to dissolve in water at different melting temperatures: above 30 °C for porcine and bovine gelatin and below 25 °C for fish-derived gelatin [16,19,29].

Differences in gelatin from different sources are also observed in the gelation temperature at which the system undergoes conformational transitions promoting the ordered reassociation of chains and leading to a partial re-establishment of the triple-helical network. Mammalian gelatin can form gels at temperatures below 25 °C, while fish gelatin presents gelation temperatures lower than 10 °C, as observed with some cold-water fish species [16,29]. These thermoreversible structures are stabilized by weak non-covalent interactions, such as hydrogen bonds oriented perpendicularly between adjacent gelatin chains, ultimately leading to gel formation [19,29]. The lower thermal stability of many fish gelatins is primarily associated with their lower proline and hydroxyproline content and has important implications for their processing and subsequent GelMA synthesis. Therefore, fish gelatin should not simply be considered as a mammalian gelatin substitute, but as a precursor with distinct molecular and thermal characteristics that may influence downstream hydrogel formation [16,33].

#### 2.4.2. Polyampholyte Behavior and Isoelectric Point

An additional relevant chemical feature of gelatin is its intrinsic polyampholyte character due to the presence of both positively and negatively charged functional groups, which makes its physicochemical behavior highly pH-responsive. At the IEP, which corresponds to the pH value at which the protein is electrically neutral, the surface charges of the gelatin molecules are balanced. Below its IEP, gelatin carries a net positive charge due to the protonation of ionizable groups, whereas above its IEP, deprotonation generates a net negative charge [29].

The pH of the medium also plays a crucial role in the structural organization of gelatin, modulating its conformational arrangement and the stability of intermolecular interactions. When the pH of the surrounding environment is close to the IEP of gelatin, the formation of helical structures is maximized, whereas deviations of the pH from this value markedly reduce the rate of helix assembly. However, as temperature decreases, the effect of the medium pH on helix formation becomes less pronounced [29].

Recent experimental work has further demonstrated that pH can significantly affect the morphology and physicochemical behavior of gelatin hydrogels, supporting the importance of the charge state of the gelatin chains and the surrounding environment in determining network organization [29].

Because Type A and Type B gelatin differ substantially in their IEP, the same environmental pH can produce different charge states and intermolecular interactions depending on the gelatin precursor [29]. This difference is therefore relevant when evaluating the subsequent behavior of Type A- and Type B-derived GelMA.

#### 2.4.3. Bloom Strength and Gel Quality

Another physicochemical parameter influencing the gel-forming properties of gelatin is the Bloom index or value, a parameter that provides a quantitative indicator of gel strength. It is determined using a Bloom gelometer, an instrument specifically designed to measure the stiffness of gelatin gels or films. The samples are prepared at a concentration of 6.67% (*w/v*) at 10 °C and are tested after 16–18 h from preparation. Processing conditions, particularly the temperature of the extraction of water, play a key role in determining the final Bloom value, which typically ranges between 50 and over 300 g [23,34].

Fish-derived gelatin generally exhibits lower Bloom numbers than many mammalian gelatins, although substantial variability exists according to fish species and habitat. Cold-water fish gelatin often exhibits lower Bloom strength than warm-water fish gelatin, but considerable variation can occur among species and extraction processes. Therefore, Bloom strength should be reported as a specific material descriptor rather than inferred from gelatin origin alone [16,35,36].

The relevance of Bloom strength extends beyond the properties of gelatin itself. Aljaber et al. demonstrated that both gelatin source and Bloom number affected the mechanical and biological properties of the resulting GelMA hydrogels, with distinct responses observed for porcine-, bovine-, and fish-derived GelMA [23]. More recently, Zambuto et al. demonstrated that gelatin Bloom strength affected not only the stiffness and viscoelasticity of GelMA hydrogels, but also their pore size, diffusivity, and permeability [24]. These findings provide direct evidence that a property of the gelatin precursor can be propagated through methacrylation and crosslinking to determine the structural, mechanical, and transport properties of the final hydrogel. This evidence supports considering Bloom strength as a relevant material descriptor when reporting and comparing GelMA formulations, particularly because two GelMA samples with similar polymer concentration and nominal degree of functionalization may still exhibit different properties if their gelatin precursors differ in Bloom strength or molecular characteristics.

### 2.5. Type A and Type B Gelatin

Type A and B gelatin exhibit distinct IEPs, approximately in the range of 6.0–9.0 and 4.7–5.4, respectively, which results in different electrostatic charges of the gelatin solution according to the pH. Type A gelatin is generally obtained through acid treatment of collagen, whereas Type B gelatin is produced through alkaline treatment, and these different processing routes contribute to differences in the isoelectric point, functional groups, molecular characteristics, and gel properties [5,10].

The molecular weight distribution and gel strength of gelatin depend not only on the gelatin type but also on the collagen source, extraction conditions, degree of hydrolysis, and manufacturing process. The α-chain of gelatin typically has a molecular weight of approximately 100–110 kDa, while β- and γ-components correspond to higher molecular weight species; therefore, a single molecular weight value should not be considered representative of either gelatin type [5,10].

Moreover, differences in gel strength between Type A and Type B gelatin are not universal and should be considered material specific. For example, comparative studies have reported a higher Bloom strength for Type A porcine gelatin than for Type B bovine gelatin, whereas other Type A/B comparisons have shown the opposite trend, highlighting the importance of reporting the gelatin source and Bloom strength rather than inferring mechanical properties solely from the gelatin type [23,36,37].

The different chemical treatments also influence the abundance of functional groups and the degree of deamidation; alkaline processing characteristic of Type B gelatin generally results in the greater conversion of amide groups into carboxyl groups, contributing to its lower isoelectric point [5].

The solubility at physiological temperature (37 °C) of both gelatin types, which results in the progressive loss of mechanical strength and structural integrity, prevents their direct use in in vivo applications. This thermal instability arises from the loss of the physical gelatin network above its gelation/transition temperature and limits the use of unmodified gelatin as a stable three-dimensional matrix at physiological temperature. Therefore, the implementation of chemical, physical, or enzymatic crosslinking strategies is generally required to stabilize gelatin-based hydrogels under physiological conditions [5,10].

### 2.6. Crosslinking Strategies for Gelatin-Based Hydrogels

Crosslinking methods significantly influence material key properties, including not only the biocompatibility profile but also degradation kinetics, mechanical properties, and rheological behavior. Gelatin-based hydrogels can be stabilized through physical, chemical, enzymatic, or combined crosslinking strategies, each providing a different balance between network stability, processability, cytocompatibility, and control over degradation and mechanical properties [5].

Physical crosslinking relies primarily on non-covalent interactions or physical and thermal treatments that stabilize the gelatin network without requiring a conventional chemical crosslinking agent. Depending on the approach, stabilization may involve hydrogen bonding, hydrophobic interactions, electrostatic interactions, or thermally induced intermolecular associations, as observed in cooling-induced gelatin gelation and dehydrothermal treatment [5,19,38].

Physical crosslinking can minimize the use of potentially cytotoxic chemical reagents and may therefore be advantageous for applications requiring high cytocompatibility. However, physically stabilized gelatin networks generally exhibit lower long-term stability and greater sensitivity to temperature and hydration conditions than covalently crosslinked systems [5,19,38].

This limitation can be particularly relevant under physiological conditions, where the reversible nature of physical interactions may compromise long-term mechanical integrity. Chemical crosslinking can overcome these limitations by generating covalent bonds between gelatin chains, producing networks with enhanced structural stability, mechanical strength, and more controllable degradation behavior [5,19,38].

Chemical crosslinking can be broadly classified according to the mechanism by which covalent bonds are introduced into the polymer network. Zero-length crosslinking promotes direct bond formation between functional groups already present on gelatin chains, as in carbodiimide-mediated coupling of carboxyl and amino groups, without incorporation of the activating reagent into the final network. Conversely, non-zero-length strategies employ bifunctional or multifunctional crosslinking molecules that become incorporated into the resulting polymer network. These approaches allow extensive modulation of network structure and mechanical properties, although their biocompatibility depends on the chemical nature of the crosslinker and the efficient removal of potentially cytotoxic residual reagents [1,5,19].

A representative example of a chemically functionalized gelatin system is GelMA, obtained through the reaction of gelatin with methacrylic anhydride (MA), which introduces methacrylamide and methacrylate functionalities that subsequently enable covalent crosslinking, typically through radical-mediated photopolymerization. The degree of functionalization (DoF) is highly dependent on the MA concentration, gelatin source, reaction conditions, and purification procedure and can therefore vary substantially among GelMA preparations [5,7].

Finally, enzymatic crosslinking represents an alternative strategy for stabilizing gelatin hydrogels through the formation of covalent bonds between polymer chains and is particularly attractive for cell-laden hydrogels because enzymatic reactions can generally be performed under relatively mild and aqueous conditions. In particular, transglutaminase catalyzes reactions between glutamine and primary amine residues, promoting network formation under mild conditions. Other enzymes, including tyrosinase and horseradish peroxidase, have also been investigated for enzymatic hydrogel formation, with the selected enzyme influencing reaction kinetics, network structure, and the resulting biological and mechanical properties [7].

## 3. Gelatin Methacryloyl: GelMA

### 3.1. Chemical Functionalization of Gelatin and GelMA Synthesis

Gelatin methacryloyl (GelMA) is obtained through the chemical functionalization of gelatin with methacryloyl groups, most commonly using methacrylic anhydride (MA). The reaction exploits the nucleophilic residues present in the gelatin backbone, primarily the ε-amino groups of lysine and hydroxylysine, as well as hydroxyl-containing residues, resulting in the introduction of methacryloyl functionalities that provide photo-crosslinkable double bonds. The extent and efficiency of this modification depend not only on the amount of MA employed, but also on the characteristics of the gelatin precursor and on the reaction environment, including pH, buffer composition, temperature, reaction time, and polymer concentration. Consequently, GelMA should not be regarded as a chemically uniform material, as apparently similar synthesis protocols may generate polymers with different degrees of functionalization and physicochemical properties [14,25,33,39].

During conventional GelMA synthesis, less than 5% of the amino acid residues of gelatin react with MA. Consequently, most functional amino acid motifs, including RGD sequences and MMP-sensitive sites involved in cell adhesion, enzymatic degradation, and ECM remodeling, are preserved, thereby retaining the intrinsic bioactivity of the gelatin precursor.

The conventional synthesis of GelMA is generally performed by dissolving gelatin in an aqueous buffer at a temperature above its gelation temperature, followed by the addition of MA under continuous stirring. The reaction is subsequently quenched or diluted, and the modified polymer is purified, commonly by dialysis, to remove unreacted MA, methacrylic acid, salts, and other low-molecular-weight reaction products. The purified GelMA is generally freeze-dried and stored until use. The original methodology introduced by Van Den Bulcke et al. [39] has subsequently been adopted and modified in numerous studies, with substantial variations in gelatin concentration, MA amount, reaction temperature, reaction time, pH, buffer composition, and purification procedures.

**Figure 2:**
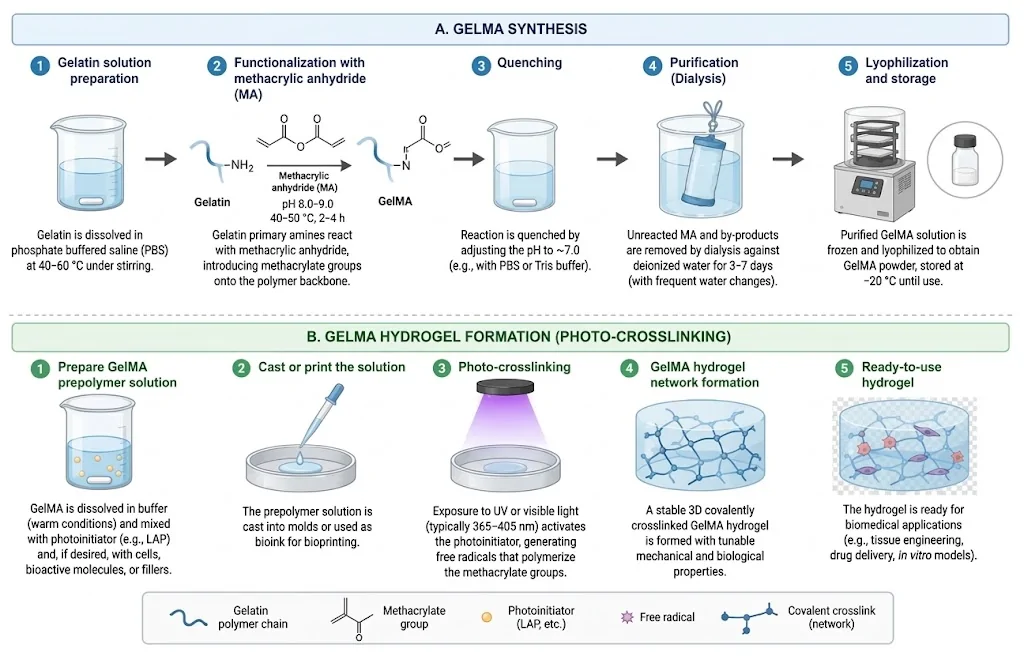
Schematic representation of the main steps involved in GelMA preparation and hydrogel formation. Gelatin is chemically functionalized with methacrylic anhydride, followed by quenching, purification by dialysis, and freeze-drying to obtain GelMA (**A**). In the presence of a suitable photoinitiator, GelMA undergoes photo-crosslinking upon light exposure, resulting in the formation of a three-dimensional covalently crosslinked hydrogel network (**B**).

Importantly, these modifications are not merely procedural differences because they can affect the chemical structure of the resulting GelMA and consequently its subsequent crosslinking behavior. Recent experimental evidence has demonstrated that the reaction system itself can influence GelMA structure and hydrogel performance. Chen et al. [40], for example, directly compared GelMA synthesized in phosphate-buffered saline (PBS) and carbonate–bicarbonate buffer solution (CBS) at comparable degrees of methacryloylation. Despite targeting similar functionalization levels, the resulting polymers exhibited differences in physical structure and in the properties of the corresponding hydrogels, including gel–sol transition temperature, photocuring efficiency, mechanical strength, swelling behavior, pore size, and porosity. The main steps involved in GelMA preparation, from gelatin methacrylation to purification and subsequent photo-crosslinking, are schematically summarized in Figure 2.

These findings indicate that the degree of methacryloylation alone cannot fully describe the chemical and functional characteristics of GelMA preparation. Rather, the relationship between synthesis conditions and the final material properties is multifactorial. In particular, the reaction environment can modify the interactions occurring within and between gelatin chains, thereby influencing the structure of the precursor polymer before and after methacrylation. Chen et al. [40] proposed that differences between PBS- and CBS-derived GelMA could be associated with changes in intrachain and interchain interactions, including hydrogen bonding, following functionalization.

The gelatin source represents an additional variable that is often insufficiently considered when comparing GelMA formulations. Gelatin obtained from different animal sources, or even different gelatin types originating from the same broad source category, may exhibit differences in molecular characteristics and available reactive groups, which can affect the efficiency of methacrylation. Gaglio et al. [25] directly investigated the combined effects of gelatin source and synthesis protocol by comparing GelMA obtained from Type A porcine and Type B bovine gelatin using two different synthetic approaches. Although the two synthesis methods produced comparable degrees of functionalization for Type A gelatin, a marked difference was observed for Type B gelatin, for which the measured DoF differed by more than 10% between the two protocols.

This observation is particularly relevant because it demonstrates that the effect of a synthesis protocol cannot necessarily be extrapolated independently of the gelatin precursor. In other words, changing the gelatin source while maintaining the same nominal methacrylation protocol does not guarantee the production of chemically equivalent GelMA. Gaglio et al. [25] further showed that these differences in functionalization were associated with changes in the physicochemical behavior of the resulting hydrogels, including rheological and mechanical properties, swelling, and enzymatic degradation, highlighting the interdependence between gelatin source, synthesis procedure, and final GelMA performance.

Recent evidence further supports this source-dependent behavior. Di Muzio et al. [33] compared GelMA derived from porcine, bovine, and fish gelatin using either a conventional biphasic synthesis or a homogeneous monophasic strategy based on DMSO. Their results demonstrated that both gelatin origin and synthetic approach significantly affected the degree of derivatization, secondary structure, thermoresponsive behavior, and rheological properties of the resulting polymers. Notably, the monophasic approach allowed finer control over the degree of derivatization but simultaneously altered the rheological behavior of GelMA, suggesting that changes in reaction conditions may influence polymer–polymer interactions beyond the extent of chemical functionalization itself.

Therefore, the synthesis of GelMA should be considered as a multivariable process in which the properties of the starting gelatin and the parameters of the methacrylation reaction jointly determine the characteristics of the final macromer. This is particularly relevant when GelMA formulations from different studies are compared, as similar nominal DoF values may be obtained from different gelatin precursors and reaction conditions without necessarily resulting in equivalent polymers or hydrogels.

The introduction of methacryloyl groups converts gelatin from a predominantly thermoreversible biopolymer into a photo-crosslinkable macromer. Upon exposure to light in the presence of a suitable photoinitiator, the methacryloyl double bonds undergo free-radical polymerization, generating a covalently crosslinked three-dimensional network. The resulting hydrogel therefore combines the biological characteristics inherited from gelatin with the possibility of controlling network formation through the degree of functionalization, polymer concentration, photoinitiator concentration, wavelength, light intensity, and irradiation time.

From this perspective, the degree of functionalization (DoF), although one of the most frequently reported parameters used to characterize GelMA, should be interpreted as one component of a broader structure–processing–property relationship rather than as an independent predictor of hydrogel performance. The number of methacryloyl groups available for polymerization determines the potential crosslinking density, but the actual network formed upon photo-crosslinking also depends on the GelMA concentration and the specific photopolymerization conditions. Thus, differences in hydrogel stiffness, swelling, degradation, or pore architecture reported between studies cannot necessarily be attributed to DoF alone.

This issue is particularly important when GelMA derived from different gelatin sources is compared. Recent studies have shown that precursor characteristics can interact with DoF to determine the final properties of the material. Ajgaonkar et al. [41], for example, investigated GelMA formulations differing in gelatin Bloom strength and degree of functionalization and demonstrated that these parameters jointly influenced mechanical stability, degradation, printability, and cellular behavior. Such findings reinforce the concept that gelatin source, precursor characteristics, degree of functionalization, and crosslinking conditions should be considered as interconnected variables rather than independent parameters.

Accordingly, a meaningful description of GelMA synthesis should report not only the nominal amount of MA and the resulting DoF, but also the origin and characteristics of the gelatin precursor, gelatin concentration, reaction buffer and pH, reaction temperature and duration, purification procedure, and method used for DoF determination. Such comprehensive reporting is essential for improving reproducibility and facilitating the comparison of GelMA formulations across different studies.

Overall, the available evidence suggests that the synthesis of GelMA is not a standardized, source-independent process. Instead, it represents a chain of interconnected variables in which the characteristics of the gelatin precursor influence its chemical functionalization, while the reaction conditions further determine the structure of the resulting macromer. These factors ultimately affect the behavior of the photo-crosslinked hydrogel and should therefore be considered when relating GelMA synthesis to its biomedical performance. The main differences between gelatin and GelMA, including their crosslinking mechanisms, thermoresponsiveness, mechanical stability, degradation behavior, printability, and principal biomedical applications, are summarized in Table 1.

### 3.2. Degree of Substitution and Degree of Functionalization

Given the biphasic nature of GelMA synthesis, in which an organic reagent is introduced into an aqueous phase, both the mixing conditions and the concentration of methacrylic anhydride (MA) employed significantly influence the dispersion quality and the resulting degree of substitution (DS). However, recent evidence indicates that the relationship between MA concentration and functionalization is not necessarily linear and cannot be considered independently of the gelatin precursor, reaction pH, buffer composition, temperature, and reaction time [25,33,42].

The DS of GelMA represents the amount of lysine groups replaced by MA during the synthesis, and it is maximized by maintaining alkaline conditions throughout the reaction. More precisely, DS describes the fraction of reactive residues that have been functionalized, whereas the degree of functionalization (DoF) is commonly used more broadly to indicate the extent of methacryloyl incorporation into the gelatin backbone. Although these terms are frequently used interchangeably in the literature, their definition and analytical calculation may vary among studies and should therefore be clearly specified when reporting GelMA properties.

To achieve a high DS, GelMA synthesis in PBS generally requires a relatively high MA concentration (approximately 10–32 molar excess in some established protocols), mainly because the formation of methacrylic acid as a reaction by-product progressively lowers the pH of the solution. This acidification shifts the system away from the IEP of gelatin, thereby reducing the availability of neutral lysine amino groups required to react efficiently with MA. Thus, the effective availability of reactive amino groups, rather than the nominal amount of MA alone, is a key determinant of methacrylation efficiency.

Recent experimental studies have provided direct evidence for this interaction between MA concentration and reaction pH. Gómez-Lizárraga et al. [42] demonstrated that increasing MA concentration did not necessarily result in a proportional increase in DoF when the reaction pH was allowed to decrease during methacrylation. In their experimental system, controlling the pH had a stronger effect on functionalization than increasing MA concentration or extending the reaction time under uncontrolled conditions. These findings indicate that maintaining a favorable reaction environment may be more effective than simply increasing the amount of methacrylation reagent.

In order to reduce the amount of MA and limit its potential cytotoxic effects, GelMA can be produced employing a carbonate–bicarbonate solution (CBS), which enables a near-complete substitution (reported DS values up to approximately 97% in specific protocols). In this case, GelMA synthesis is performed using a modified protocol based on the sequential addition of MA (approximately 2 molar excess), with periodic pH adjustment of the reaction mixture to the optimal buffer value at 30-min intervals [43,44].

The importance of the reaction system extends beyond its effect on DS. Chen et al. [40] demonstrated that GelMA synthesized in PBS and carbonate–bicarbonate buffer can exhibit different gel–sol transition temperatures, photocuring efficiency, mechanical properties, swelling behavior, pore size, and porosity, even when the materials present comparable degrees of functionalization. This observation suggests that the buffer system can affect the molecular organization and intermolecular interactions of GelMA independently of the nominal DoF.

Accordingly, PBS and CBS should not be considered simply interchangeable reaction media. Differences in ionic environment, pH and buffering capacity can affect both the chemical reaction and the subsequent physicochemical behavior of the modified polymer. This is consistent with the observation that GelMA produced in PBS showed higher gel–sol transition temperatures, improved photocuring efficiency, and enhanced mechanical properties, whereas GelMA synthesized in CBS exhibited greater swelling and larger pores with increased porosity.

#### 3.2.1. Effect of Methacrylic Anhydride Concentration

The amount of MA used during GelMA synthesis is one of the main parameters used to modulate the final degree of functionalization. Increasing the MA-to-gelatin ratio generally increases the number of methacryloyl groups introduced into the polymer. However, this relationship depends on the chemical environment and on the characteristics of the gelatin precursor.

Gaglio et al. [25] demonstrated this source-dependent behavior by comparing Type A porcine and Type B bovine gelatin. Increasing the MA amount increased the DoF in both gelatin types, but the relationship between reagent availability and functionalization differed according to the precursor and synthesis protocol. Their results therefore indicate that the amount of MA required to achieve a specific target DoF cannot necessarily be transferred from one gelatin source to another.

This point is particularly relevant because a similar nominal MA concentration does not guarantee an equivalent degree of functionalization. Conversely, similar DoF values can be obtained using different MA concentrations when other reaction parameters are modified. Therefore, reporting the MA-to-gelatin ratio, rather than MA concentration alone, is preferable when comparing GelMA synthesis protocols.

#### 3.2.2. Effect of pH and Buffer System

The reaction pH is a critical parameter because methacrylation generates methacrylic acid as a by-product, progressively acidifying the reaction mixture. Maintaining an alkaline environment favors the availability of unprotonated amino groups and consequently facilitates their reaction with MA.

Gómez-Lizárraga et al. [42] experimentally confirmed that pH control can substantially influence GelMA functionalization and that increasing the reaction time or MA concentration cannot necessarily compensate for uncontrolled acidification. This provides a mechanistic explanation for the long-standing observation that CBS-based protocols can achieve high DS using lower MA excess than conventional PBS-based procedures.

The buffer system can also influence the properties of the final GelMA independently of DS. Chen et al. [40] showed that the reaction medium affected the molecular and physicochemical characteristics of GelMA, indicating that the effects of pH and buffer composition extend beyond the chemical conversion itself.

#### 3.2.3. Influence of Gelatin Precursor Characteristics

The characteristics of the gelatin precursor represent another major determinant of GelMA functionalization. Gelatin source, Type A or Type B classification, molecular-weight distribution, Bloom strength, amino acid composition, and IEP can affect the availability and reactivity of the groups involved in methacrylation. Consequently, the same synthetic protocol may result in different DoF values depending on the gelatin used.

Gaglio et al. [25] directly demonstrated this source dependence by comparing Type A porcine and Type B bovine gelatin. Importantly, their results showed that the difference between the two gelatin types was not constant but depended on the amount of MA available during the reaction. At high MA feed ratios, the two gelatin types could reach comparable DS values, whereas differences became more evident at lower reagent availability.

This observation is particularly relevant to the interpretation of GelMA literature because it demonstrates that precursor effects may become more pronounced when the reaction is performed under conditions of limited MA availability. Thus, gelatin source and reaction conditions should be considered as interacting variables rather than independent parameters.

Di Muzio et al. [33] further extended this concept by comparing GelMA derived from porcine, bovine, and fish gelatin and demonstrating that both gelatin origin and synthetic strategy influenced the degree of derivatization, thermoresponsive behavior and rheological properties. This recent evidence supports the need to report the characteristics of the starting gelatin when describing or comparing GelMA materials.

#### 3.2.4. Effect of Reaction Temperature and Time

Reaction temperature and time also contribute to the efficiency of gelatin methacrylation. Gelatin must be maintained in a solubilized state during the reaction, and temperatures commonly used for GelMA synthesis are therefore above the thermoreversible gelation temperature of the precursor. However, increasing reaction temperature or duration does not necessarily result in a proportional increase in DoF.

Gómez-Lizárraga et al. [42] showed that extending the reaction time from 1 to 3 h under uncontrolled pH conditions did not overcome the reduction in functionalization associated with reaction acidification. This finding further supports the concept that reaction time must be considered together with pH and MA availability rather than as an independent parameter.

Similarly, Gaglio et al. [25] demonstrated that changes in the overall synthetic protocol, including reaction conditions and MA addition strategy, could substantially affect the final DoF, particularly when comparing different gelatin types. More recently, Di Muzio et al. [33] confirmed that changes in reaction strategy can alter both derivatization efficiency and the rheological behavior of GelMA derived from different gelatin sources.

#### 3.2.5. Determination of DS and DoF

DS of GelMA is most commonly quantified by ^1^H-NMR spectroscopy, by integrating the characteristic spectral peaks associated with methacryloyl groups and comparing them with appropriate signals from unmodified gelatin. This approach provides a direct chemical characterization of methacryloyl incorporation and is generally considered the most informative method for determining the extent of functionalization [36,42,44]. However, differences in NMR acquisition and calculation procedures can contribute to variability among reported values.

In addition to ^1^H-NMR, colorimetric methods can be employed. The 2,4,6-trinitrobenzenesulfonic acid (TNBS) assay quantifies the concentration of amino groups, whereas the ninhydrin assay indirectly evaluates the extent of methacrylation by measuring residual free amine groups after the reaction [14,42,44,45]. Because these approaches quantify residual amino groups rather than methacryloyl groups directly, values obtained using different analytical methods should not automatically be considered interchangeable.

The quantity of MA used during GelMA synthesis influences, but does not by itself define, the DoF of the biomaterial. GelMA formulations are frequently classified as low-, medium-, or high-DoF materials, with representative values around 60%, 85%, and 100%, respectively; however, these categories should be regarded as practical descriptors rather than universally standardized classes [25].

The DoF is an important determinant of the subsequent crosslinking behavior because a greater number of methacryloyl groups provides a higher theoretical number of sites available for radical polymerization. Consequently, increasing DoF can generally increase network crosslinking density and reduce swelling. Nevertheless, the final properties of the photo-crosslinked hydrogel also depend on polymer concentration, photoinitiator concentration, light wavelength, irradiation intensity, and exposure time. Therefore, DoF should not be considered an isolated predictor of hydrogel mechanics or swelling behavior.

Higher DoF values generally lead to increased crosslinking density, resulting in tighter polymeric networks characterized by decreased swelling rates. This relationship provides the basis for using DoF as a design parameter to tune GelMA hydrogel properties, but the recent literature indicates that its effect is strongly modulated by the precursor gelatin and by the subsequent photo-crosslinking conditions [24,25,33,42].

#### 3.2.6. Overall Considerations

Taken together, the available evidence indicates that GelMA functionalization should be considered as the outcome of an interconnected synthesis process rather than as a direct consequence of MA concentration alone. MA availability, pH and buffer composition, gelatin source and characteristics, temperature and reaction time collectively determine the extent of methacrylation and the molecular characteristics of the resulting GelMA.

This is particularly important for the comparison of literature data, as apparently similar DoF values may be obtained using different gelatin precursors and reaction conditions and may therefore correspond to polymers with different physicochemical properties. Conversely, GelMA formulations with different DoF values may exhibit comparable macroscopic behavior when differences in gelatin source, polymer concentration, or crosslinking conditions compensate for the variation in functionalization.

For this reason, a complete description of GelMA synthesis should include the gelatin source and type, gelatin concentration, MA-to-gelatin ratio, reaction pH, buffer composition, temperature, reaction time, purification procedure, analytical method used for DoF determination, and the resulting experimentally measured DoF. Such reporting is essential to improve reproducibility and to establish meaningful relationships between GelMA chemistry, hydrogel structure, and biomedical performance.

### 3.3. GelMA-Based Hydrogels

#### 3.3.1. Photo-Crosslinking Mechanism

The introduction of methacrylate groups enables gelatin to undergo photo-crosslinking, leading to the formation of covalently crosslinked and transparent hydrogels, which are particularly suitable for microscopic evaluation. The photo-polymerization process can take place under mild conditions (neutral pH, room temperature, aqueous environment, etc.) and consists in a free radical reaction that occurs in the presence of a photoinitiator such as Lithium phenyl-2,4,6-trimethylbenzoylphosphinate (LAP) in an aqueous state [14,46].

Ultraviolet (UV) irradiation of the photoinitiator brings to the formation of free radicals through homolytic cleavage that starts chain-growth polymerization. After this phase, chain propagation takes place among methacrylate groups and terminates between a propagating chain and another free radical [14,46]. Compared to other methods, photo-polymerization has proven to be the most suitable approach for a spatiotemporal controlled generation of hydrogels. Through photo-induced covalent crosslinking of methacrylate groups, GelMA develops a stable polymer network at physiological temperatures, making it particularly attractive for the fabrication of 3D constructs in TE [44,47,48,49,50]. A major advantage of GelMA lies in its ability to couple tunable mechanical properties with the intrinsic biological features of gelatin, while providing enhanced structural stability. Like gelatin, GelMA preserves a highly hydrated microenvironment that is essential to support cell adhesion, growth, and proliferation [25,50].

The efficiency of GelMA photo-crosslinking, however, is not determined solely by the presence of methacrylate groups. It depends on the combined characteristics of the GelMA macromer and the photopolymerization system, including degree of functionalization, polymer concentration, photoinitiator type and concentration, irradiation wavelength, light intensity, exposure time, and oxygen availability [14]. Therefore, the same GelMA formulation may exhibit markedly different crosslinking behavior when processed under different photopolymerization conditions [3].

The mechanical and physical properties of GelMA-based hydrogels are largely governed by formulation and processing conditions, as GelMA concentration, DS, and photo-polymerization parameters (photoinitiator concentration, UV light intensity, and exposure time) can be modified to meet specific application requirements [3]. Through precise control of these variables, GelMA-based hydrogels can be engineered with defined architecture and predictable properties, enabling the optimization of both mechanical stability and biological response.

Photoinitiators can be broadly classified into Type I and Type II systems according to their radical-generation mechanism [51]. Type I photoinitiators undergo direct photochemical cleavage to generate radicals, whereas Type II systems require an additional co-initiator or electron/hydrogen donor [14,51]. This distinction is relevant for GelMA because the activation wavelength, radical-generation efficiency, and cytocompatibility of the photoinitiator determine the balance between curing kinetics and cellular exposure [51].

Irgacure 2959 (I2959), lithium phenyl-2,4,6-trimethylbenzoylphosphinate (LAP), Eosin Y (EY), and ruthenium/sodium persulfate (Ru/SPS) represent commonly investigated photoinitiating systems for GelMA. Their activation conditions and cytocompatibility differ substantially, making photoinitiator selection application dependent rather than interchangeable. Sharifi et al. [12] demonstrated that systematic optimization of visible light-induced GelMA crosslinking requires consideration of both photoinitiator concentration and irradiation conditions, particularly when cell compatibility is a major constraint.

I2959 is a well-established Type I photoinitiator commonly activated using UV light around 365 nm. However, its relatively limited absorption at this wavelength may require longer irradiation times or higher concentrations, potentially increasing the exposure of encapsulated cells to UV radiation and photogenerated radicals. LAP has consequently become an increasingly attractive alternative because of its efficient activation in the UV/violet range and its relatively rapid polymerization kinetics [52].

In a direct comparison of I2959 and LAP in cell-laden GelMA-based hydrogels, Xu et al. [52] reported that both photoinitiators maintained relatively good cell viability at 0.3 and 0.5% (*w/v*) during the printing process. However, at higher concentrations of 0.7 and 0.9% (*w/v*), LAP resulted in markedly better cell viability than I2959. After photo-crosslinking, this difference was no longer evident, with comparable post-printing viability observed for the two systems [46]. This suggests that photoinitiator-related cytotoxicity may depend not only on the irradiation step itself, but also on the duration of cellular exposure to the uncrosslinked formulation.

I2959 has been extensively used for GelMA crosslinking in many biomedical applications because of its established protocols and relatively good cytocompatibility at low concentrations. However, its dependence on UV irradiation and comparatively limited curing efficiency reduces its suitability for in situ applications, where prolonged irradiation may damage encapsulated cells and surrounding tissues. Consequently, I2959 remains particularly useful for conventional in vitro hydrogel fabrication, whereas its translational potential for minimally invasive or deep-tissue applications is more restricted [52].

LAP, by contrast, can provide faster gelation at relatively low concentrations and can be activated at wavelengths closer to the visible range, although its cytocompatibility remains dependent on concentration and light dose. Duymaz et al. [14] further demonstrated that the photoinitiation process itself can substantially influence the crosslinking behavior of GelMA networks, reinforcing the need to consider photoinitiator chemistry and irradiation conditions together rather than as independent parameters. Compared to other methods, photo-polymerization has proven to be the most suitable approach for a spatiotemporal controlled generation of hydrogels. Overall, photo-crosslinking represents a major advantage of GelMA because it enables spatial and temporal control over network formation; however, the choice of photoinitiator and irradiation parameters must balance polymerization efficiency, network properties, penetration depth, and cytocompatibility.

Table 2 summarizes the key characteristics of the main photoinitiating systems used for GelMA crosslinking, including activation mechanism and wavelength, curing efficiency, cytocompatibility, and main advantages and limitations.

#### 3.3.2. Mechanical and Rheological Properties

The mechanical properties of GelMA-based hydrogels are governed by both the characteristics of the polymer network and the experimental conditions under which they are assessed. GelMA concentration, DS, gelatin source, and Bloom strength, as well as photo-crosslinking conditions, can substantially modify hydrogel stiffness. However, the mechanical values reported for these hydrogels also depend on the testing methodology, loading mode and experimental protocol. Consequently, parameters such as compressive modulus, tensile Young’s modulus, rheological storage and loss modulus (G′ and G″, respectively), dynamic storage and loss modulus (E′ and E″, respectively), and indentation-derived elastic modulus should not be considered directly interchangeable.

GelMA-based hydrogels’ mechanical properties as stiffness, elastic Young’s modulus, and adhesive strength are directly proportional to GelMA concentration; samples prepared at 5% *w/v* exhibit elastic Young’s modulus values of 1.55 kPa while the same samples realized at 15% *w/v* present values of 48.50 kPa [53,54]. As the GelMA concentration increases, the density of methacrylate polymer chains within the solution also rises, strengthening intermolecular interactions such as van der Waals forces and hydrogen bonding, while increasing the probability of crosslinking between neighboring methacrylate groups during photoinitiated radical polymerization. As a consequence, a denser and more tightly interconnected covalent network is formed [39,55].

While GelMA concentrations below 5% *w/v* are generally insufficient to ensure stable hydrogel formation, concentrations equal to or higher than 15% *w/v* have been shown to improve mechanical properties of the resulting hydrogel [53,54]. However, increasing GelMA concentration does not exclusively affect mechanical stiffness: it may also modify pore size, porosity, molecular transport, swelling behavior, degradation kinetics, and cellular responses [7,53]. Therefore, a higher polymer concentration should not be interpreted universally as an improvement in hydrogel performance, but rather as a formulation parameter that must be optimized according to the intended application [48,53].

The increase in GelMA concentration and the subsequent rise in matrix stiffness is not sufficient to achieve the same mechanical performances as synthetic hydrogels. For this reason, GelMA is often combined with other polymers, such as polyethylene glycol (PEG), polyacrylic acid (PAA), alginate, or with nanomaterials like graphene oxide and carbon nanotubes to enhance its properties and electrical conductivity [23,44].

Other synthesis parameters that are directly proportional to matrix rigidity are both the DS and the polymer mass/volume fraction; accordingly, the compressive modulus of GelMA-based hydrogels increases with methacrylic substitution, reaching 2.0 ± 0.18 kPa at 49.8% DS, 3.2 ± 0.18 kPa at 63.8% DS, and 4.5 ± 0.33 kPa at 73.2% DS [7,56].

The DS affects both the organization of the crosslinked network and the conformational behavior of the polymer chains. At higher DS, the introduction of methacrylate groups reduces chain flexibility and promotes a more compact and ordered molecular arrangement, thereby increasing resistance to deformation. By contrast, lower DS is associated with looser molecular packing and greater structural rearrangement under mechanical stress [53]. Similarly, the Bloom value of the starting GelMA also plays a relevant role in determining the rigidity of the resulting hydrogel. Lower Bloom indexes have been associated with reduced stiffness and viscoelasticity, as well as lower material purity, consistent with the trends observed for the parent gelatin [24].

Importantly, the effect of DS on stiffness should be interpreted together with GelMA concentration and precursor characteristics. A high DoF increases the theoretical number of crosslinkable methacryloyl groups, but the final network density depends on how many of these groups effectively participate in photopolymerization. Thus, DoF alone does not fully predict the mechanical response of the final hydrogel.

GelMA-based hydrogels’ stiffness also depends on photo-polymerization settings, as crosslinking agent concentration and exposure time. Higher photoinitiator concentrations lead to the formation of a greater number of propagating sites, resulting in shorter polymer chains and increased crosslinks within the hydrogel network [14,20,25]. However, this behavior is not universally observed. Notably, LAP has been reported to promote the formation of hydrogels with enhanced mechanical properties even at relatively low concentrations, while also enabling faster photo-polymerization because of its higher polymerization kinetics [13,46].

Regarding exposure time, it shows a sigmoidal correlation with hydrogel tensile properties which increase as a function of time, until reaching plateau trends at the end of the crosslinking reaction. This indicates that, once the gelation process is complete, additional UV exposure does not further enhance the mechanical performance of the hydrogel [13].

Recent work has reinforced the need to distinguish between photoinitiator concentration, irradiation intensity, and irradiation time. Duymaz et al. [14] showed that changes in the photoinitiation process can modify GelMA network formation, while Sharifi et al. [12] demonstrated that visible light crosslinking conditions require systematic optimization to achieve an appropriate balance between curing efficiency and cell compatibility.

Matrix stiffness represents a key design parameter in GelMA-based systems, as it not only defines the mechanical behavior of the hydrogel but also determines how closely the material can mimic the properties of native tissues. The accurate characterization and modulation of GelMA hydrogels stiffness are essential for the development of biomaterials intended for TE and regenerative medicine applications. The rigidity of the hydrogel has been shown to directly regulate cellular behaviors, including adhesion, spreading, proliferation, and stem cell differentiation, all of which are fundamental processes in tissue repair and regeneration [57].

Regarding the rheological properties of GelMA, the main parameters considered are the storage modulus (G′) and the loss modulus (G″), both of which generally increase with increasing GelMA concentration. These parameters are typically evaluated through rheological and photo-rheological analyses, which provide information on the viscoelastic behavior of the material by monitoring the evolution of G′ and G″ over time. Specifically, G′ reflects the elastic component of the hydrogel and thus its ability to store deformation energy, whereas G″ describes the viscous component, corresponding to the energy dissipated during deformation. In GelMA-based systems, the sol–gel transition is identified by the crossover point between G′ and G″, after which the elastic contribution becomes dominant [20,57].

The sol–gel transition and the resulting viscoelastic response are influenced by several interacting parameters, including reaction temperature, GelMA concentration, degree of functionalization, photoinitiator concentration, crosslinking conditions, and gelatin origin. Consequently, rheological measurements should be interpreted together with the formulation and processing conditions rather than as intrinsic, formulation-independent properties of GelMA [14].

These parameters collectively determine the density, organization, and stability of the crosslinked polymer network and, consequently, the mechanical response of the resulting hydrogel. In Table 3 are summarized the main formulation and processing parameters affecting the mechanical and rheological properties of GelMA-based hydrogels [36,58].

While the parameters summarized in Table 3 determine the formulation- and processing-dependent mechanical and rheological behavior of GelMA hydrogels, the values reported in the literature are also strongly influenced by how these properties are experimentally measured. Different testing techniques probe distinct deformation modes, length scales, and timescales and therefore capture different aspects of the hydrogel response [20]. Accordingly, differences in reported mechanical values cannot always be attributed solely to differences in GelMA formulation, since methodological variables may contribute substantially to the observed variability.

For this reason, mechanical and rheological parameters obtained using different techniques should be interpreted within the context of the experimental protocol used to obtain them. An important aspect in the interpretation of GelMA mechanical properties is the testing methodology employed, as different techniques, including compression testing, tensile testing, rheology, dynamic mechanical analysis (DMA), and nanoindentation, probe distinct deformation modes, length scales, and time-dependent responses. The main characteristics of these approaches, together with the parameters measured and the principal factors affecting the reported values, are summarized in Table 4.

Compression testing is among the most used methods and provides a bulk assessment of the hydrogel response to compressive loading, typically through the determination of a compressive modulus from a selected region of the stress–strain curve. However, the resulting values are influenced by the strain range used for modulus calculation, loading rate, sample geometry, hydration state, and boundary conditions. Tensile testing similarly provides a bulk measurement but under elongational loading, allowing the determination of Young’s modulus, tensile strength, and strain at failure. Tensile and compressive moduli therefore should not be considered directly interchangeable [20]. Indeed, Torza et al. recently characterized hollow cylindrical GelMA-based hydrogels under tension, compression, and shear using a triaxial loading protocol. Three loading cycles were applied sequentially up to 10% compressive strain, 10% tensile strain, and between −20% and +20% shear strain. The results revealed a clear tension–compression asymmetry, with the peak compressive stress being approximately 16% higher than the peak tensile stress, while cell incorporation produced the largest asymmetry of approximately 19% [59]. A possible explanation for this behavior lies in the different response of the hydrated polymer network to compression and tension, together with the structural changes introduced by cell incorporation. A higher cell density can reduce the effective GelMA volume fraction and crosslinking density while increasing matrix porosity, thereby disrupting the continuity of the polymer network. These changes may affect load transfer differently under tensile and compressive deformation, thus contributing to the greater tension–compression asymmetry observed in cell-laden constructs [7,53].

Oscillatory rheology provides complementary information by characterizing the viscoelastic response of GelMA under shear deformation. As described above, G′ and G″ quantify the elastic and viscous contributions, respectively, but their absolute values depend on experimental parameters such as strain amplitude, oscillation frequency, temperature, and measuring geometry. Photo-rheology further enables these parameters to be monitored during light exposure, providing information on the evolution of the network during crosslinking. In a recent study, Ribezzi et al. monitored both G′ and G″ at 1 Hz and 1% strain during GelMA photo-crosslinking. The final G′ varied markedly with molecular weight, degree of modification, and polymer concentration, ranging from 379 ± 47 Pa for 5% (*w/v*) 90 kDa GelMA with 40% modification to 171.46 kPa for 20% (*w/v*) 160 kDa GelMA with 80% modification. The relative evolution of G′ and G″ also provided information on structural transitions of the material: following high shear deformation, G′ recovered to values higher than G″, indicating restoration of a predominantly elastic, solid-like network, while a G′–G″ crossover was observed at approximately 480% strain during a strain sweep, corresponding to a transition towards liquid-like behavior [13]. These results indicate that rheological parameters should be interpreted in relation to both network composition and testing conditions rather than as intrinsic material constants. The marked increase in G′ observed with higher molecular weight, degree of modification, and GelMA concentration can be attributed to the greater number of polymer chains and reactive groups available for network formation, which promotes a denser and more interconnected crosslinked structure. At the same time, the relationship between G′ and G″ provides additional information on network stability: the predominance of G′ after crosslinking reflects the formation of a mainly elastic network, whereas the G′–G″ crossover at high strain indicates progressive disruption of this structure and a transition towards a more viscous response. Differences in G′ and G″ across studies may arise not only from GelMA formulation but also from variations in strain amplitude, frequency, temperature, and crosslinking conditions, limiting direct comparison of rheological values obtained under different experimental protocols.

DMA can be used to investigate the time-dependent mechanical response of GelMA-based hydrogels under different loading configurations. In oscillatory axial tests, DMA provides the values of E′ and E″, which describe the elastic and viscous components of the material response, respectively. These parameters are analogous to G′ and G″ obtained from oscillatory shear rheology, although they are measured using different deformation modes and should therefore not be directly compared. Keskin-Erdogan et al. characterized GelMA hydrogels under sinusoidal uniaxial compression over 0.05–20 Hz at 0.1% strain, thereby assessing their mechanical response over different deformation timescales. For GelMA crosslinked for 60 s, E′ and E″ were 24.73 ± 2.52 and 1.08 ± 0.23 kPa, respectively, with E′ remaining higher than E″ across the investigated frequency range, indicating a predominantly elastic response [30]. The marked predominance of E′ over E″ indicates that the GelMA network responds mainly elastically under the investigated loading conditions, with relatively limited viscous energy dissipation. This behavior can be attributed to the covalently crosslinked polymer network, which constrains chain mobility and favors elastic recovery during cyclic deformation. However, the reported E′ and E″ values remain frequency- and strain-dependent and should therefore not be considered intrinsic material constants. Their comparison across studies requires similar loading amplitudes, frequency ranges, sample geometries, and crosslinking conditions.

DMA can also be used for non-oscillatory measurements, including quasi-static compression and stress-relaxation tests. While compression provides information on hydrogel stiffness, stress-relaxation is a time-dependent test in which the material is held at a constant deformation and the decrease in stress is monitored over time, providing information on its viscoelastic response. In the study of Ribezzi et al., for example, 5% (*w/v*) GelMA formulations were characterized using both approaches. In compression, the modulus increased with molecular weight and degree of modification: for 90 kDa GelMA, it rose from 0.40 ± 0.04 to 0.96 ± 0.10 kPa as the degree of modification increased from 40% to 80%, whereas 160 kDa formulations showed values between 1.50 ± 0.18 and 8.90 ± 0.58 kPa over the same range of modification. Stress-relaxation measurements showed that the residual stress index increased from 37.8% for 90 kDa GelMA with a degree of modification of 40% to 89.8% for 160 kDa GelMA with a degree of modification of 80% [60]. This indicates that lower molecular weight and less functionalized networks relaxed stress more rapidly, whereas higher molecular weight and highly functionalized GelMA retained a greater proportion of the applied stress over time.

On a smaller scale, indentation techniques, particularly atomic force microscopy (AFM)-based nanoindentation, provide local measurements of hydrogel stiffness and can reveal mechanical heterogeneities that may not be detected by bulk testing. However, the resulting indentation modulus depends on probe geometry, indentation depth and rate, sample thickness, and the contact model used to analyze the force–indentation curves. Minopoli et al. investigated the rate-dependent response of GelMA-based hydrogels using AFM nanoindentation with indentation velocities ranging from 0.5 to 15 μm/s, an 8 nm-radius probe, and a maximum force of 5.5 nN. The apparent Young’s modulus of GelMA increased non-linearly from approximately 15–16 kPa at low indentation rates to around 18 kPa at 15 μm/s, demonstrating that the measured nanoscale stiffness depends on the deformation timescale. This rate-dependent stiffening can be explained by the viscoelastic nature of the polymer network: at lower indentation rates, polymer chains have more time to rearrange and relax, whereas rapid deformation limits these processes and produces a higher apparent stiffness. Accordingly, purely elastic models such as the Hertz model may not fully describe GelMA mechanics, as viscous contributions can influence the modulus obtained from AFM measurements. Minopoli et al. therefore applied viscoelastic models to separate elastic and viscous contributions to the measured response [32]. Overall, nanoindentation-derived values should be compared cautiously with bulk measurements, since they are influenced not only by GelMA formulation and crosslinking conditions but also by the local scale and rate at which the material is probed.

Taken together, these considerations highlight that the mechanical and rheological properties reported for GelMA are not solely material-intrinsic parameters, but are also dependent on the formulation, crosslinking history, deformation mode, measurement scale, and experimental timescale.

Each testing method captures a different aspect of GelMA mechanics. Compression and tensile tests assess bulk behavior under distinct loading modes, rheology and oscillatory DMA describe viscoelastic responses across defined timescales, stress-relaxation quantifies time-dependent stress dissipation, and nanoindentation probes local mechanical properties at smaller length scales. Combining these approaches therefore provides a more complete characterization of GelMA mechanical behavior and, importantly, improves the interpretation and comparability of data reported across different studies (Table 4).

#### 3.3.3. Swelling, Porosity and Degradation

The main physical properties of GelMA-based hydrogels include swelling behavior, matrix pore size, and degradability, as these features play a central role in determining the overall performance of the biomaterial. In particular, they influence not only the structural organization and stability of the hydrogel network, but also the transport of water, nutrients, oxygen, and therapeutic molecules within the matrix, which is particularly relevant for cell survival, drug delivery, and TE applications.

Swelling behavior is a key property of hydrogel networks, as it influences solute diffusion, surface characteristics, mechanical performances, and structural fidelity. In GelMA hydrogels, the swelling degree is strongly affected by both the GelMA concentration and DS. Specifically, at a fixed polymer concentration, the mass swelling ratio increases as the DS decreases, indicating a greater capacity of the network to absorb and retain water. Conversely, at a fixed DS, lower polymer concentrations are associated with higher swelling ratios. Overall, higher GelMA concentrations and greater DS reduce the swelling capacity of the hydrogel by increasing matrix rigidity, thereby limiting water uptake as well as the diffusion of nutrients and oxygen. At the same time, these conditions improve the structural fidelity of the resulting hydrogel architecture, particularly in micropatterned constructs [25,55].

This inverse relationship between crosslinking density and swelling should nevertheless be interpreted in the context of the complete hydrogel formulation. GelMA concentration and DoF influence network density, but swelling is also affected by ionic strength, pH, degradation, crosslinking efficiency, and the characteristics of the precursor gelatin. Therefore, differences in swelling ratios between studies cannot necessarily be attributed to DoF alone.

Swelling capability of GelMA hydrogels is commonly assessed by a gravimetric method. Briefly, hydrogels are prepared under the desired experimental conditions, freeze-dried, and weighed to determine their initial dry mass. The samples are then immersed in a swelling medium, typically ultrapure water or PBS at pH 7.4, and reweighed at predetermined time intervals. This procedure allows evaluation of the swelling equilibrium and calculation of the swelling ratio according to the following equation [55]:

Swelling ratio = [(wet weight − dry weight)/dry weight] × 100

Matrix porosity is governed by the main GelMA synthesis parameters, particularly polymer concentration and DS. Together, these variables play a decisive role in defining pore size, which in GelMA-based hydrogels generally ranges from 50–100 μm for micropores to 200–700 μm for macropores. Higher DS are associated with smaller pore sizes and increasing GelMA concentration has been shown to reduce both the overall porosity and the average pore size of the hydrogel network [55].

However, reported pore-size values should be interpreted cautiously because pore architecture is highly dependent on the fabrication method and characterization technique. In particular, drying procedures used before SEM analysis can alter the hydrated network and may produce apparent pore dimensions that differ from those of the hydrogel in its physiological, fully hydrated state.

Pore architecture represents a critical parameter, particularly in biomedical applications, since the lack of a vascular network makes pore size and distribution essential factors governing the diffusion of oxygen and nutrients within the hydrogel, as well as the removal of metabolic waste products from the matrix. Accordingly, high porosity and an appropriate pore size are favorable for cell culture, as they support mass transport and facilitate cell ingrowth, proliferation, migration, and differentiation. Moreover, when GelMA hydrogels are used as in vitro pathological models for drug screening, pore size must be carefully controlled to ensure efficient transport of therapeutic agents throughout the matrix [44,55]. In addition to formulation parameters, porosity can also be modulated by UV light intensity during photo-polymerization, as this influences free-radical generation and reaction kinetics. Specifically, lower UV intensity leads to reduced radical formation and slower crosslinking, favoring the development of more homogeneous macrostructures, whereas higher intensity promotes faster radical generation and intramolecular cyclization, resulting in microgel-like architectures, although potentially at the expense of cell viability [44]. This observation further emphasizes that pore architecture should be considered a processing-dependent property rather than a direct consequence of GelMA concentration or DoF alone. In particular, photopolymerization kinetics can alter network heterogeneity and therefore affect both pore dimensions and transport properties.

GelMA hydrogels may undergo relatively rapid degradation under aqueous and physiological-like conditions, due to the retainment of functional amino acid motifs from native gelatin and collagen, including MMP sequences. Studies have shown that the degradation of these hydrogels is strongly influenced by both the DoF and the polymer concentration, with lower functionalization generally associated with faster degradation.

This property is commonly evaluated by monitoring changes in hydrogel weight over time following incubation in a selected medium, often containing degradative enzymes such as type I and type II collagenase, also known as MMP-1 and MMP-8, respectively. In a typical protocol, hydrogels are incubated at 37 °C, periodically collected, gently blotted to remove excess surface liquid, freeze-dried, and then weighed to determine the residual mass. The degradation degree is subsequently calculated as the percentage of mass loss relative to the initial weight. In addition, the degradation kinetics can be further modulated by the concentration and type of enzyme present in the surrounding environment [55].

Importantly, enzymatic degradation assays should be interpreted as model systems rather than direct representations of in vivo degradation. The enzyme type, concentration, incubation time, hydrogel geometry, DoF, polymer concentration, and crosslinking efficiency can all substantially affect the measured degradation rate. Therefore, degradation data obtained using different enzyme systems or experimental conditions should not be directly compared without considering these variables.

Overall, swelling, porosity and degradation are closely interconnected with the network structure generated during GelMA photo-crosslinking. Higher polymer concentration and DoF generally favor denser networks, resulting in reduced swelling, smaller pores, and slower degradation, whereas lower crosslinking density generally produces more hydrated and permissive networks. However, these relationships are not absolute because precursor characteristics, photopolymerization conditions, and degradation environment can substantially modify the final behavior [7].

The main formulation and processing parameters affecting GelMA hydrogel properties are summarized in Table 5.

### 3.4. Influence of Gelatin Type: Type A vs. Type B GelMA

Both Type A and Type B gelatin can be successfully functionalized to produce GelMA, yielding a cell-compatible material. However, the distinction between these two types of GelMA deserves specific attention, since the properties of the precursor gelatin represent a major source of variability in the synthesis and final performance of this biomaterial. Direct quantitative comparisons between Type A and Type B GelMA remain relatively limited, and the available results are strongly dependent on synthesis and testing conditions. Nevertheless, the existing studies demonstrate that the gelatin type together with the functionalization can affect IEP, DS, viscoelasticity, rheological behavior, mechanical properties, swelling, printability, degradation, and cell viability.

Importantly, however, Type A versus Type B should not be considered as a binary predictor of GelMA performance. The available evidence indicates that the behavior of the resulting hydrogel emerges from the interaction between gelatin pretreatment and source, precursor characteristics such as Bloom strength and molecular-weight distribution, degree of functionalization, polymer concentration, synthesis protocol, and photo-crosslinking conditions. Recent studies have therefore shifted the interpretation from a simple “Type A versus Type B” comparison toward a broader precursor–processing–property relationship [23,24,25,33]. The characteristics of the precursor gelatin represent a major source of variability in GelMA synthesis and final performance. This distinction is particularly relevant because apparently contradictory results reported for Type A and Type B GelMA may arise from differences in the experimental conditions rather than from an intrinsic superiority of one gelatin type.

#### 3.4.1. Isoelectric Point of Type A vs. Type B GelMA

As previously discussed, the fundamental difference between Type A and Type B gelatin lies in the different pre-treatment processes applied to the starting material, acidic and alkaline, respectively. This distinction is also closely related to the source of the raw material, as porcine and fish gelatin are generally produced through acid treatment, whereas bovine gelatin is more commonly obtained through alkaline processing. These pre-treatment conditions influence both the chemical and physical properties of the resulting gelatin, including amino acid composition, IEP, viscosity, and, consequently, also affect the characteristics of the derived GelMA. Alkaline processing promotes the conversion of asparagine and glutamine residues of the native collagen into aspartic acid and glutamic acid, whereas acidic treatment largely preserves these amino acids. Consequently, Type A gelatin exhibits a higher IEP than Type B, leading to opposite charge behavior under physiological conditions [22].

Methacrylation markedly decreases the IEP of both Type A and Type B gelatin; however, a difference between the corresponding GelMA derivatives is generally maintained. This behavior is primarily related to the functional groups involved in both the original gelatin composition and the derivatization process. During GelMA synthesis, MA reacts mainly with the free primary amine groups of lysine and hydroxylysine residues, although a limited reaction with hydroxyl motifs may also occur. The functionalization therefore reduces the number of protonatable groups, which are responsible for positive charges, while the carboxyl groups of acidic amino acids, such as aspartic acid and glutamic acid, which account for negative charges, remain essentially unaltered. As a result, the balance between positively and negatively ionizable groups shifts toward a more acidic profile, leading to a decrease in IEP after methacrylation. Since Type B gelatin contains a higher relative abundance of acidic carboxyl-containing residues than Type A gelatin, this difference persists even after modification, so that Type B GelMA generally retains a lower IEP than Type A GelMA [22].

This theoretical prediction was quantitatively confirmed by the study of Sewald et al. The unmodified Type A and Type B gelatins exhibited markedly different experimental IEP values of 8.8 and 4.9, respectively, in agreement with the calculated values of 9.1 and 4.7. Following methacrylation, the IEP decreased significantly for both gelatin types, reaching approximately 4.5 for Type A GelMA and 4.1 for Type B, with the difference between the two derivatives remaining statistically significant (*p* < 0.01). These experimental values were also closely reproduced by theoretical estimates, which predicted IEPs of 4.7 and 4.2 for Type A and Type B GelMA, respectively. The smaller shift observed for Type B GelMA is consistent with gelatin initially higher content of acidic side chains and excess negative charges. Overall, the close agreement between predicted and experimentally measured IEP values supports the proposed mechanism whereby methacrylation consumes positively charged amino groups while leaving negatively charged carboxyl groups largely unaffected, thereby preserving a lower IEP in Type B GelMA even after functionalization.

The persistence of a lower IEP in Type B GelMA is relevant because charge distribution influences polymer–polymer and polymer–water interactions and may consequently contribute to differences in swelling, rheological behavior, and cellular interactions. However, IEP alone cannot account for the differences observed between Type A and Type B GelMA, which are also affected by DoF, polymer concentration and the molecular characteristics of the precursor.

#### 3.4.2. Degree of Substitution of Type A vs. Type B GelMA

Despite the intrinsic compositional differences between Type A and Type B GelMA, comparable DoFs can generally be achieved when the same MA-to-gelatin feed ratio is used. However, the extent of this similarity depends on the amount of methacrylic anhydride available during the reaction. In the study conducted by Lee B. et al., Type A and Type B GelMA exhibited virtually identical DS values at the highest feed ratio tested, 0.1 mL MA/g gelatin, reaching 94.9 ± 0.5% and 94.9 ± 0.6%, respectively. By contrast, at a feed ratio of 0.05 mL MA/g gelatin, Type B GelMA showed a significantly higher DS than Type A GelMA, with values of 82.7 ± 1.5% and 72.6 ± 0.6%, corresponding to an absolute difference of 10.1 percentage points. Smaller but still evident differences were also observed at lower feed ratios, with DS values of 45.7 ± 3.5% versus 39.5 ± 1.8% at 0.025/1 and 20.7 ± 6.4% versus 14.8 ± 0.8% at 0.0125/1 for Type B and Type A GelMA, respectively. These results suggest that gelatin type has a limited impact on functionalization when high amounts of MA are used, whereas its influence becomes more apparent as reagent availability decreases. Even when similar levels of functionalization are achieved, Type A and Type B GelMA may still exhibit distinct physicochemical properties, including differences in viscoelasticity, G’ and swelling behavior [10].

Recent evidence further supports the need to interpret these differences in relation to the synthesis protocol. Gaglio et al. [25] demonstrated that the DoF achieved in Type A and Type B gelatin depended not only on the amount of MA used but also on the synthetic strategy, with the magnitude of the difference between the two gelatin types changing according to the protocol. Similarly, Di Muzio et al. [33] showed that gelatin origin and reaction strategy jointly affected the degree of derivatization, further indicating that precursor type cannot be separated from the conditions under which methacrylation is performed.

These findings are particularly relevant to the interpretation of DoF. A comparable DoF does not necessarily imply chemically or physically equivalent GelMA because the precursor gelatin may retain differences in molecular weight, amino acid composition, charge distribution, and chain organization. Conversely, differences in reported DoF between Type A and Type B GelMA may partly reflect differences in reaction efficiency rather than intrinsic differences in the functional behavior of the resulting networks.

#### 3.4.3. Viscosity and Shear-Thinning Behavior of Type A vs. Type B GelMA

Both Type A and Type B GelMA exhibit shear-thinning behavior, meaning that their viscosity decreases as the applied shear rate increases. Their response is mostly modulated by the type of precursor gelatin, the DS, and the polymer concentration [10,25]. The study conducted by Lee et al. [10] showed that, at 37 °C and a concentration of 30% (*w/v*), increasing DS resulted in lower viscosity and shear stress for both GelMA types.

Nevertheless, at comparable concentration and DS, Type A GelMA consistently exhibited lower overall viscosity and shear stress than Type B GelMA. This difference suggests that methacrylation does not eliminate the physicochemical characteristics inherited from the parent gelatin. The distinct amino acid composition, charge distribution at physiological temperature, and intermolecular interactions associated with the acid and alkaline treated precursors may influence chain association in solution and thereby contribute to the higher resistance to flow observed for Type B GelMA. A different effect emerged when the measurements were performed at 25 °C, where polymer concentration became a major determinant of flow behavior.

At this temperature, 30% (*w/v*) formulations formed physical gels and consequently no longer displayed the typical shear-thinning profile. When the concentration was reduced to 20% (*w/v*), however, highly functionalized Type A and Type B GelMA exhibited comparable pseudoplastic characteristics over a broad shear-rate range of 1–1000 s^−1^. Moreover, Type A GelMA presents a more pronounced temperature-dependent viscosity profile than the Type B one. These findings indicate that gelatin type and DS contribute to the differences in viscosity of the two hydrogels’ formulations observed under physiological conditions, while the rheological response at lower temperatures is also strongly governed by polymer concentration and the onset of physical gelation.

These observations illustrate why rheological differences between Type A and Type B GelMA should not be interpreted as an intrinsic consequence of the gelatin type alone. The effect of the precursor becomes evident only within a specific formulation and temperature window, while polymer concentration and the extent of functionalization can substantially modify or even mask the contribution of gelatin type. This distinction is particularly relevant for bioink applications, in which viscosity and shear-thinning behavior must be evaluated under the temperature, concentration, and shear-rate conditions relevant to the intended printing process.

#### 3.4.4. Storage Modulus and Recovery Rate of Type A vs. Type B GelMA

In addition to affecting viscosity, the type of starting gelatin also influences the sol–gel transition and the final G′ of the hydrogel.

Gaglio et al. [25] reported higher G′ values for Type A than for Type B GelMA under specific high DoF conditions, with values of approximately 31.6 ± 2.0 kPa and 27.7 ± 1.3 kPa, respectively. Conversely, Lee et al. [10] observed the opposite trend, with Type B GelMA reaching a G′ of 53.1 ± 0.7 kPa, compared with 34.7 ± 2.4 kPa for Type A GelMA. These contrasting quantitative findings indicate that the viscoelastic response of GelMA cannot be attributed to the gelatin type alone, but rather reflects the combined influence of precursor source, Bloom strength, DoF, polymer concentration, synthesis protocol, and crosslinking conditions. Gelatin type should therefore be considered as one of several interacting parameters governing the final rheological behavior of the hydrogel.

Differences were also observed in rheological recovery after deformation. Type B GelMA recovered 73%, 90%, and 98% of its initial viscoelastic properties for high-, medium-, and low-DoF formulations, respectively, whereas Type A GelMA restored only approximately 40% within the first 20 s [25]. Photorheological analysis further demonstrated a clear dependence of G′ on DoF for both GelMA types, with higher functionalization levels corresponding to higher G′ values. These observations reinforce the view that the final viscoelastic behavior of GelMA emerges from the interplay between precursor characteristics and formulation parameters rather than from the Type A and Type B distinction alone.

The opposite trends reported by Gaglio et al. and Lee et al. [10,25] therefore represent an important finding rather than a contradiction to be resolved by assigning superior mechanical properties to one gelatin type. Instead, they demonstrate that Type A/Type B classification is insufficient to predict G′ without considering the complete formulation and testing conditions. In particular, Bloom strength, DoF, polymer concentration and crosslinking conditions may act as confounding or interacting variables and should be reported when comparing the mechanical behavior of different GelMA preparations.

#### 3.4.5. Bloom Number of Type A vs. Type B GelMA

The Bloom value of the parent gelatin is an important parameter to consider, as it reflects the gel strength of the starting material and can directly influence the mechanical properties of the resulting GelMA.

However, Bloom strength should not be inferred directly from the gelatin type. Rather, it is a material-specific descriptor influenced by gelatin source, extraction and processing history, molecular-weight distribution, and batch characteristics. Aljaber et al. [23] demonstrated that both gelatin source and Bloom number influenced the mechanical and biological properties of GelMA hydrogels, while Zambuto et al. [24] further showed that the Bloom strength of the starting gelatin affected the mechanical, viscoelastic, structural, and transport properties of the resulting GelMA networks.

These findings are particularly important when comparing Type A and Type B GelMA because differences attributed to “gelatin type” may in fact reflect differences in the Bloom strength of the starting materials. Therefore, Bloom strength should be reported as an independent precursor characteristic rather than used as a surrogate for Type A or Type B classification.

#### 3.4.6. Mechanical Properties of Type A vs. Type B GelMA

The mechanical performance of Type A and Type B GelMA is strongly influenced by the characteristics of the precursor gelatin, although the specific contribution of gelatin type is often intertwined with Bloom strength, molecular-weight distribution, DoF, and crosslinking conditions. This is evident from compression-based measurements; Aljaber M. et al. reported a compressive strength of approximately 60 kPa for Type B GelMA, compared with about 40 kPa for Type A GelMA prepared from 300-Bloom gelatin and 18 kPa for the corresponding 175-Bloom formulation [23]. Although these data suggest marked source-dependent differences in mechanical resistance, they cannot be attributed exclusively to the Type A and Type B distinction because gelatin source and Bloom strength varied simultaneously [60].

Indeed, these data provide a useful example of why direct Type A versus Type B comparisons require careful control of precursor characteristics. The substantially different mechanical responses observed among the formulations cannot be interpreted as evidence that Type B GelMA is intrinsically stronger than Type A GelMA because Bloom strength and gelatin source were not held constant. Aljaber et al. [23] instead demonstrated that the properties of the gelatin precursor can substantially contribute to the final mechanical behavior of GelMA, supporting the concept that precursor specification should be treated as a critical material parameter.

More broadly, mechanical properties should be interpreted as an emergent characteristic of the entire GelMA network, determined by precursor characteristics, DoF, polymer concentration, crosslinking density and photopolymerization conditions. Consequently, the same gelatin type may generate hydrogels with markedly different mechanical properties depending on how the material is functionalized and crosslinked.

#### 3.4.7. Swelling Behavior of Type A and Type B GelMA

Swelling behavior appears to differ systematically between Type A- and Type B-derived GelMA, suggesting that the characteristics inherited from the precursor gelatin contribute to water uptake independently of the degree of functionalization. In the direct comparison by Gaglio et al. [25], Type B GelMA consistently showed higher swelling than Type A GelMA at the same polymer concentration (10% (*w/v*)). Depending on the formulation, Type B hydrogels reached swelling ratios of approximately 1190–1768%, whereas Type A GelMA ranged from about 724 to 1158%. Thus, even when prepared and tested under comparable conditions, Type B formulations absorbed substantially more water than Type A counterparts. This difference may be related to the distinct chemical composition and charge distribution of the two precursor gelatins, with Type B containing a higher proportion of acidic residues and therefore exhibiting different polymer–water and intermolecular interactions.

The higher swelling observed for Type B GelMA is consistent with the differences in precursor charge distribution and IEP discussed above. However, swelling is also highly sensitive to DoF, polymer concentration, and network density. Therefore, the quantitative difference reported by Gaglio et al. [25] should be interpreted as a formulation-specific observation rather than as a universal property of all Type B GelMA.

This distinction is important because swelling affects nutrient and oxygen transport, solute diffusion, mechanical stability, and degradation and therefore may have direct consequences for the suitability of GelMA formulations in tissue engineering, drug delivery, and three-dimensional cell culture.

#### 3.4.8. Printability of Type A vs. Type B GelMA

The different rheological profiles of Type A- and Type B-derived GelMA translate into distinct processing requirements during extrusion-based bioprinting. Rather than showing a clear superiority of one gelatin type, the available studies indicate that each formulation requires specific printing conditions to achieve stable filament deposition and shape retention. Lee B. et al. demonstrated that both Type A and Type B GelMA can be successfully extruded when formulation parameters are appropriately adjusted, identifying highly functionalized 20% (*w/v*) formulations as suitable candidates for printing [10]. Gaglio et al. instead showed that the two GelMA types require markedly different thermal processing windows, with optimized printing temperatures of approximately 20 °C for Type A and 12 °C for Type B GelMA [25]. These differences are particularly relevant from a manufacturing perspective, as the use of identical printing settings for both materials may lead to suboptimal filament formation or construct stability. Moreover, low DoF Type B formulations showed limited suitability for printing because of poor post-fabrication stability, whereas high DoF formulations of both types provided more reliable construct formation [25]. The choice between Type A and Type B GelMA for bioprinting should be accompanied by type-specific optimization of processing parameters rather than by the application of a single standardized printing protocol.

These observations further support the view that gelatin type should be considered as one component of a formulation-specific processing window rather than as a criterion for selecting an intrinsically superior GelMA. In particular, the interaction between precursor properties, DoF, polymer concentration, and temperature determines the balance between pre-crosslinking flow behavior and post-deposition structural stability.

For bioprinting applications, reporting gelatin type without the corresponding Bloom strength, DoF, concentration, and printing temperature therefore provides insufficient information to reproduce the reported printability.

#### 3.4.9. Degradation of Type A vs. Type B GelMA

The degradation kinetics of Type A and Type B GelMA have rarely been directly compared. Gaglio et al. investigated this aspect by analyzing the two GelMA types produced through different synthesis protocols, evaluating not only the effect of gelatin type but also the contribution of the preparation method to enzymatic stability. Degradation was assessed in collagenase XI (0.75 U/mL) at 37 °C by monitoring hydrogel residual mass after 4, 6, 8, and 24 h. Although most formulations showed broadly comparable degradation profiles during the first 8 h, differences became more evident after prolonged incubation. One Type B GelMA underwent complete degradation within 24 h, whereas the corresponding Type A formulation and the other GelMA samples obtained through the alternative synthesis method remained partially intact [25].

The degradation behavior cannot be attributed to gelatin type alone, since the synthesis process can modify the resulting DoF, network architecture, and consequently, the susceptibility of the hydrogel to enzymatic digestion. The major contribution of DoF on hydrogel degradation was further demonstrated by Pepelanova et al.; after 3 h of collagenase exposure, a moderately functionalized GelMA formulation (A70) exhibited approximately 45% degradation at 20 U/mL and 75% at 30 U/mL of collagenase, whereas the highly functionalized formulation (A100) showed only about 20% degradation under comparable conditions. Moreover, A70 was completely digested within approximately 6 h, while A100 required up to 8 h for complete degradation even at 20 U/mL collagenase [48].

These findings illustrate the difficulty of assigning degradation behavior directly to gelatin type. Enzymatic degradation depends on the accessibility of gelatin-derived peptide sequences, network density and architecture, DoF, polymer concentration, and the specific protease and concentration used in the assay. Consequently, the higher degradation observed for one formulation cannot necessarily be extrapolated to all Type A or Type B GelMA materials.

The comparison also reinforces the concept introduced in Section 3.3: DoF is a chemical descriptor that influences, but does not uniquely determine, the effective crosslink density and degradability of the final hydrogel.

#### 3.4.10. Cellular Viability in Type A vs. Type B GelMA

From a biological perspective, the distinction between Type A and Type B GelMA appears to be less pronounced than the differences observed in their physicochemical and rheological properties. Both formulations generally provide a permissive environment for cell survival, as they retain the cell-adhesive and enzymatically degradable motifs inherited from gelatin. Quantitative data support this overall similarity; Lee et al. reported cell viabilities of approximately 75% for both Type A and Type B GelMA-based hydrogels at 20% (*w/v*), indicating comparable short-term cytocompatibility under matched conditions [10]. More recently, Gaglio et al. also compared Type A and Type B GelMA formulations with different DoFs and synthesis protocols. In high DoF constructs, cell viability remained above 85% for both GelMA types, further supporting the overall cytocompatibility of both formulations. Differences became more evident in low-DoF formulations, where Type B GelMA showed reduced construct stability because of greater swelling and degradation, whereas cells encapsulated in low-DoF Type A GelMA tended to escape from the hydrogel and proliferate on the surrounding surface [25]. These observations suggest that, although both Type A and Type B GelMA are generally suitable for cell encapsulation and culture, the most appropriate formulation may depend on the specific cell type and on the microenvironmental conditions required to support its survival, proliferation, and spreading.

Importantly, the available evidence does not support the conclusion that Type A or Type B GelMA is intrinsically more biocompatible. Rather, the cellular response appears to depend strongly on the physical stability, swelling, degradation and mechanical properties of the specific formulation. Thus, differences in cell viability may be secondary to differences in network properties rather than a direct consequence of gelatin type itself.

#### 3.4.11. Critical Comparison of Type A and Type B GelMA

The quantitative evidence discussed above demonstrates that Type A and Type B GelMA do not exhibit a universal hierarchy of performance. Some differences appear relatively consistent, particularly those related to IEP, swelling, and processing behavior, whereas the reported trends in G′, mechanical strength, and degradation are more dependent on the experimental system. This variability is not merely a limitation of the available literature; it provides evidence that gelatin type interacts with other material and processing parameters.

Accordingly, the most appropriate interpretation is not that Type A GelMA is intrinsically superior to Type B GelMA, or vice versa, but that the two precursor types can generate distinct material-property landscapes under specific synthesis and crosslinking conditions. This distinction is particularly relevant for biomedical applications, where the optimal GelMA formulation should be selected according to the desired mechanical, transport, degradation, and biological characteristics rather than according to gelatin type alone.

Recent studies support this more integrated interpretation. Aljaber et al. [23] demonstrated that gelatin source and Bloom number affect GelMA mechanical and biological properties, Gaglio et al. [25] showed that the gelatin type interacts with synthesis protocol and DoF, while Zambuto et al. [24] demonstrated the influence of Bloom strength on the mechanical, viscoelastic and structural properties of GelMA. More recently, Di Muzio et al. [33] further showed that both gelatin origin and reaction strategy influence GelMA derivatization and rheological behavior. Together, these studies indicate that the precursor should be considered a critical design variable that acts upstream of GelMA functionalization and hydrogel network formation.

This source–functionalization–network relationship represents an important consideration for the standardization of GelMA. Reporting only “Type A” or “Type B” is insufficient to define the material. Gelatin source, Bloom strength, molecular characteristics, DoF, polymer concentration, photoinitiator, and crosslinking conditions should be reported together to enable meaningful comparison between formulations. Table 6 summarizes representative quantitative data reported for Type A- and Type B-derived GelMA under the specific experimental conditions indicated. The reported values should not be interpreted as universal material constants because GelMA properties depend on the gelatin source and precursor characteristics, Bloom strength, degree of functionalization/substitution, polymer concentration, synthesis protocol, photoinitiator, and crosslinking conditions. Differences between studies, including opposite trends in storage modulus and mechanical strength, highlight the limited comparability of Type A and Type B GelMA when formulation and testing parameters are not fully standardized. Therefore, Type A or Type B classification alone should not be considered a sufficient predictor of GelMA performance.

**Table 6:** Comparative properties of Type A and Type B GelMA reported in the literature.

Property	Type A GelMA	Type B GelMA	Experimental Conditions/Basis of Comparison	Interpretation and Limitations	Ref.
**Isoelectric point (IEP)**	~4.5 after methacrylation	~4.1 after methacrylation	Type A and Type B gelatin-derived GelMA; IEP experimentally determined after methacrylation	Type B retained a lower IEP, consistent with the higher content of acidic residues inherited from alkaline pretreatment. The difference was primarily related to precursor chemistry.	[22]
**Degree of substitution (DS)**	94.9 ± 0.5% at 0.1 mL MA/g gelatin; 72.6 ± 0.6% at 0.05 mL MA/g	94.9 ± 0.6% at 0.1 mL MA/g gelatin; 82.7 ± 1.5% at 0.05 mL MA/g	Different MA feed ratios	Differences between Type A and Type B became more evident at lower MA availability, whereas very high MA feed ratios resulted in comparable functionalization.	[10]
**Viscosity/shear-thinning**	Lower viscosity and shear stress than Type B under selected physiological-temperature conditions	Higher viscosity and shear stress than Type A under comparable conditions	30% (*w/v*), 37 °C; behavior also dependent on DS and temperature	Type B exhibited greater resistance to flow, but concentration, DS, and temperature strongly modulated the response.	[10]
**Storage modulus, G′**	~31.6 ± 2.0 kPa	~27.7 ± 1.3 kPa	High-DoF formulations; rheological testing	Type A showed higher G′ in this experimental system. The result should not be generalized because formulation and synthesis conditions influence network formation.	[25]
**Storage modulus, G′**	34.7 ± 2.4 kPa	53.1 ± 0.7 kPa	Comparable formulation conditions in the study	Type B showed higher G′ in this study, illustrating that the mechanical ranking of Type A vs. Type B is not universal.	[10]
**Rheological recovery**	~40% recovery within 20 s under the reported conditions	~73%, 90%, and 98% recovery for high-, medium-, and low-DoF formulations, respectively	Recovery after deformation	Type B showed faster recovery in the reported system; however, recovery depended strongly on DoF.	[25]
**Swelling**	~724–1158% at 10% (*w/v*)	~1190–1768% at 10% (*w/v*)	Same polymer concentration; Type A vs. Type B comparison	Type B exhibited greater water uptake, potentially related to differences in charge distribution and network characteristics. Swelling was also strongly affected by DoF and crosslink density.	[25]
**Compressive strength**	~18–40 kPa depending on gelatin/Bloom formulation	~60 kPa	Different gelatin sources and Bloom strengths	The apparent superiority of Type B could not be attributed to gelatin type alone because the precursor source and Bloom strength varied simultaneously.	[23]
**Bloom strength**	Up to ~300 Bloom in the reported material	Variable; should not be inferred from Type B classification alone	Parent gelatin specification	Bloom strength was an independent precursor descriptor and should be reported separately from Type A/Type B classification.	[23,24]
**Printability**	Optimal printing temperature ~20 °C in the reported formulation	Optimal printing temperature ~12 °C in the reported formulation	Extrusion-based printing; formulation-specific conditions	Different processing windows indicated that identical printing conditions cannot necessarily be applied to both GelMA types.	[25]
**Degradation**	Generally retained partial mass after prolonged collagenase exposure in the reported comparison	One Type B formulation underwent complete degradation after 24 h	Collagenase XI, 0.75 U/mL, 37 °C	Differences emerged mainly at longer incubation times and were influenced by the synthesis protocol and DoF.	[25]
**Cell viability**	>85% for high-DoF formulations; ~75% under selected 20% (*w/v*) conditions	>85% for high-DoF formulations; ~75% under selected 20% (*w/v*) conditions	Cell encapsulation/culture under study-specific conditions	Both types generally showed good cytocompatibility. Differences in biological performance may arise indirectly from differences in swelling, degradation, and network stability.	[10,25]

## 4. Biomedical Applications of GelMA-Based Hydrogels

Among its many biomedical applications, GelMA-based hydrogels have attracted considerable interest in TE, drug delivery, and the development of in vitro models [61]. Owing to polymer unique combination of biocompatibility, bioactivity, and tunable physicochemical properties, GelMA hydrogels provide a favorable microenvironment for cell encapsulation and tissue development [2]. In TE, their ability to mimic the ECM and support cell adhesion, proliferation, and differentiation makes these hydrogels a powerful platform for the fabrication of three-dimensional constructs [62]. In the context of drug delivery instead, the porous and hydrated network of GelMA-based hydrogels enables controlled loading and release of therapeutic agents [2]. Furthermore, the adjustable mechanical and structural properties of these hydrogels allow the development of physiologically relevant in vitro models, which are increasingly used to study cell behavior, disease progression, and drug response [2,57,63,64].

### 4.1. GelMA-Based Hydrogels in Tissue Engineering

TE is an interdisciplinary field aimed at restoring, replacing, or regenerating damaged tissues through the integration of cells, biomaterials, and biochemical or physicochemical cues. It has emerged as a promising alternative to conventional strategies such as organ transplantation, mechanical devices, and surgical reconstruction, which are often limited by donor shortage, risk of rejection, incomplete functional recovery, or long-term complications. A central goal of TE is the development of three-dimensional constructs able to recreate key features of the native ECM, thereby providing a more physiologically relevant environment for cell growth, migration, and differentiation than traditional two-dimensional (2D) culture systems. In this context, tissue-engineered platforms are not only valuable for regenerative medicine, but also represent a promising alternative to animal models, as they can provide more relevant human-specific environments while reducing ethical and experimental limitations [49].

GelMA-based hydrogels have been extensively investigated for the regeneration of a wide range of tissues, including skin, cartilage, bone, and blood vessels, and have also shown considerable potential in cardiovascular and neural applications. By modulating synthesis parameters such as the DS and the polymer concentration, together with the possibility of controlling degradation behavior, GelMA hydrogels are particularly well suited for skin regeneration. In this context, the integration of GelMA with advanced fabrication approaches, as 3D bioprinting, is further expanding its potential in regenerative medicine and wound healing, enabling the development of customized wound dressings and scaffolds with improved structural and biological functionalities [50,61].

#### 4.1.1. Skin Tissue Engineering and Wound Healing

GelMA-based hydrogels derived from porcine, bovine, and fish sources have been employed in skin-related applications. Zhao X. et al. proposed porcine GelMA-based hydrogels with tunable mechanical and degradation properties, achieved by varying polymer concentration. These hydrogels exhibited high cell viability and supported keratinocyte adhesion, proliferation, and differentiation, while also promoting their stratification into a multilayered epidermis with functional barrier properties [65]. Similarly, fish-derived GelMA hydrogels also support cells growth, thanks to the presence of omega-3 fatty acids, which can play a complementary role by promoting the proliferation of keratinocyte and enhancing epidermal barrier formation through related signaling pathways [17]. Moreover, these fatty acids can be converted into bioactive mediators involved in the clearance of pathogens, dead cells, and inflammatory tissue debris. They can also stimulate macrophage phagocytic activity and facilitate bacterial membrane disruption, thereby contributing as an additional defense mechanism against microbial infection [17,66].

Antimicrobial activity is particularly relevant in the context of wound healing, where the injury site is highly susceptible to bacterial contamination and subsequent infection. In the study conducted by Jahan I. et al., this function was achieved by incorporating silver nanoparticles into bovine-derived GelMA hydrogels. This combination was shown to promote faster wound closure by coupling enhanced cell migration with antibacterial activity, highlighting its potential as an effective platform for wound healing applications [66].

The most suitable GelMA formulation for skin-related applications varies according to the specific functional requirements of the intended use. Unmodified GelMA is generally advantageous when biocompatibility, cell adhesion, and matrix remodeling represent the main design priorities [2]. Conversely, when antimicrobial protection is a key requirement, greater benefits may be achieved through the incorporation of antibacterial agents, such as metallic nanoparticles [67,68], or using GelMA derived from biological sources naturally associated with potentially bioactive constituents. These strategies may provide additional antimicrobial or anti-inflammatory functionality; however, their effectiveness depends on the preservation, concentration, and biological availability of such components in the final hydrogel formulation [69]. Moreover, the introduction of exogenous antibacterial agents requires careful optimization to balance antimicrobial efficacy with cytocompatibility and tissue integration [67,68].

#### 4.1.2. Cartilage Tissue Engineering

To reproduce the properties of specific native ECM, the tunability of GelMA-based hydrogels is essential. This is particularly evident in the case of cartilage ECM, where the ability to modulate matrix stiffness is of crucial importance, as cartilage is characterized by distinct mechanical requirements that strongly influence cell behavior and tissue functionality. Alongside mechanical features, morphological properties, especially pore size, are also of critical relevance and should be tailored to the specific cellular population involved. Indeed, several studies have shown that pore sizes equal to or greater than 300 μm can promote the differentiation of mesenchymal stem cells into chondrocytes, while ensuring efficient cell distribution, nutrient transport, and adequate oxygen availability. In contrast, smaller pore sizes, typically in the range of 150–250 μm, are more suitable for supporting the growth of articular chondrocytes [70].

Despite the possibility of modulating its mechanical properties by varying the polymer concentration or DoF, GelMA still exhibits lower mechanical strength than synthetic polymers, which restricts its application in highly load-bearing sites, including articular joints. To overcome this limitation, hybrid formulations incorporating reinforcing nanomaterials or additional structural polymers have been developed to enhance the mechanical performance of GelMA-based hydrogels. In this regard, several studies have highlighted the effectiveness of such strategies in overcoming the intrinsic mechanical weakness of GelMA and expanding its potential for cartilage repair. For instance, Visser J. et al. combined Type A porcine GelMA with polycaprolactone (PCL), achieving a mechanical resistance up to 54-fold higher than that of GelMA alone. In another study, Chen J. et al. developed a ternary hydrogel system based on Type B bovine GelMA, methacrylated PEG, and heparin, designed to guide the chondrogenic differentiation of bone marrow-derived mesenchymal stem cells and to support the maintenance of their cartilaginous phenotype [71,72].

Fish-derived GelMA can also be employed in the field of cartilage TE, as many studies have shown that fish-based biomaterials are able to support chondrogenic differentiation by upregulating cartilage-specific markers and promoting ECM deposition. In addition, the bioactive components naturally present in fish-derived materials, as antimicrobial peptides and omega-3 fatty acids, contribute to maintaining cell viability and matrix integrity, creating a favorable microenvironment for cartilage formation and joint tissue repair [73,74]. A promising candidate for cartilage TE is the matrix proposed by Machado I. et al. in which fish GelMA is combined with previously methacrylated glycosaminoglycans, namely hyaluronic acid and chondroitin sulphate, to obtain photo-crosslinked hybrid hydrogels with adjustable physicochemical properties, high biocompatibility, and improved chondrocyte viability [75].

These systems represent complementary design strategies, as they differ in composition, architecture, crosslinking conditions, and intended function. Consequently, their overall performance cannot be directly compared without considering the specific requirements of the target application.

Formulations in which GelMA is combined with reinforcing synthetic polymers can be particularly advantageous when high mechanical resistance and load-bearing performance are required. GelMA–PCL represents a notable example of this strategy, as the highly organized fiber architecture of the PCL scaffold redistributes mechanical loads and constrains the lateral expansion of the hydrogel during compression, allowing the composite to reproduce stress–strain curves comparable to those of healthy articular cartilage [71]. Similar reinforcing frameworks based on other synthetic polymers may be adopted for the same purpose. However, excessively dense architectures may reduce the space available for tissue formation and restrict water transport, nutrient diffusion, cell migration, and ECM deposition; therefore, fiber organization, porosity, and polymer concentration must be carefully optimized.

Other multicomponent formulations are instead designed primarily to regulate the physicochemical and biological microenvironment rather than to maximize mechanical reinforcement. In GelMA-PEG-heparin hydrogel, PEG contributes to the structural and stress-relaxation behavior of the network, gelatin provides cell-responsive cues, and heparin modulates the presentation of bioactive molecules. Compared with GelMA alone, exhibited a weaker relaxation modulus, indicating a less mechanically constrained and more stress-relaxing matrix that may facilitate cell-mediated remodeling, molecular transport, and extracellular matrix deposition. The hydrogel supported mesenchymal stem cell chondrogenesis and displayed anti-osteogenic behavior, as indicated by the absence of mineral staining and the lack of biologically meaningful expression of collagen type X and bone sialoprotein [72].

When greater biochemical biomimicry of the native cartilage ECM is required, GelMA can be combined with cartilage-relevant macromolecules that promote a highly hydrated microenvironment and introduce tissue-specific biochemical cues. In these formulations, GelMA provides cell-adhesive and matrix-remodeling motifs, whereas the additional ECM-derived components enhance the biochemical similarity to native cartilage and may support chondrocyte viability, phenotype maintenance, and extracellular matrix production [75].

#### 4.1.3. Bone Tissue Engineering

Regarding to bone TE, which aims to restore or regenerate damaged bone, key requirements include adequate mechanical strength, osteoconductivity, and the ability to support cell viability, differentiation, and vascularization. As in other TE applications, the most common strategy adopted to improve the mechanical properties of GelMA-based hydrogels in bone regeneration is their combination with synthetic polymers. For instance, in the study conducted by Wang Y. et al., bovine-derived GelMA is functionalized with diacrylate PEG, obtaining hybrid hydrogels with enhanced mechanical stability and more controlled degradation behavior [76]. Compared with pure GelMA, GelMA-PEGDA hydrogels exhibited markedly improved mechanical performance and maintained their structural integrity for more than four weeks, a feature particularly advantageous for in vivo implantation. In vitro studies further demonstrated that osteoblasts were able to adhere and proliferate on the hydrogel surface, confirming the good cytocompatibility and biocompatibility of the hybrid system [76].

When GelMA is applied in stem cell-based bone regeneration instead, additional challenges must be considered, since mechanical stress during injection may reduce cell retention and damage cell membranes, while the lack of a supportive 3D structure can compromise cell viability and function after transplantation. To address these limitations, Zhao X. et al. proposed a microsphere system based on porcine GelMA for the encapsulation of bone marrow-derived mesenchymal stem cells and growth factors. This platform was shown to support cell survival, proliferation, migration, and osteogenic differentiation, thereby promoting bone formation [77]. Not only bovine- and porcine-derived GelMA but also fish-derived GelMA has been investigated for bone TE applications. In this case, the interest is mainly related to the presence of bioactive components capable of supporting osteogenesis through different mechanisms. Omega-3 fatty acids contribute to the regulation of the inflammatory microenvironment and promote osteoblast differentiation and mineralization. Antimicrobial peptides instead support this process by activating specific signaling pathways that enhance cell survival and proliferation, while also providing protection against infection. Finally, hydroxyapatite plays a key role in promoting osteogenic differentiation and mineral deposition, favoring bone matrix formation [17]. To further enhance the biomimetic features of the hydrogel, Huang L. et al. incorporated nano-sized fish bone into GelMA-based hydrogels, developing a composite system capable of improving mechanical performance while modulating the immune microenvironment. This combined effect contributes to improve bone regeneration, highlighting the potential of these biomimetic hydrogels as advanced materials with osteo-immunomodulatory properties [78].

The comparison among GelMA-based systems for bone TE highlights a progressive shift from structural stabilization to cell delivery and biological stimulation. The combination of GelMA with synthetic crosslinkable polymers can improve network stability, regulate degradation, and prolong scaffold integrity, thereby addressing the limited mechanical durability of unmodified GelMA. GelMA–PEGDA hydrogels illustrate this approach, with PEGDA forming a more stable polymeric network that enables the construct to retain its structural stability over extended periods. Similar effects may potentially be achieved using other compatible synthetic polymers or crosslinkable macromers capable of reinforcing the GelMA network and modulating its degradation profile [44]. However, these components mainly provide physicochemical and structural improvements and do not necessarily confer direct osteogenic activity; consequently, they may require association with cells, growth factors, or mineral phases to actively promote bone formation.

Microsphere-based GelMA systems adopt a different approach by prioritizing injectability and the localized delivery of cells and growth factors. Their geometry facilitates molecular exchange and cell–matrix interactions, but their discontinuous structure generally provides less mechanical support than reinforced bulk scaffolds [77].

Mineral-containing composites further extend the functionality of GelMA by introducing an inorganic phase that more closely resembles native bone. The incorporation of hydroxyapatite-rich particles, including nano-sized fish bone components, can enhance osteoconductivity, promote mineral deposition, and improve the osteogenic potential of the construct. However, the mineral content and particle dispersion must be carefully optimized, as aggregation or sedimentation may compromise the uniformity and reproducibility of the resulting hydrogel [79].

Taken together, these formulations should be regarded as complementary rather than directly interchangeable, as each addresses a distinct limitation of GelMA in bone regeneration.

#### 4.1.4. Vascular and Lymphatic Tissue Engineering

The viability of engineered tissues is often constrained by the difficulty in reproducing the complex architecture of native organs, particularly the vascular networks responsible for the transport of nutrients, oxygen, and metabolic waste. Consequently, the establishment of functional vascularization within engineered constructs represents a fundamental requirement. A major challenge in this context is the ability to mimic the native ECM while simultaneously promoting the formation of organized and functional vascular structures. GelMA-based hydrogels are widely employed for this purpose, owing to their intrinsic biological properties, which derive from the retention of bioactive motifs that regulate cell adhesion, proliferation, and organization into vascular networks.

As first demonstrated by Chen Y. et al., GelMA hydrogels can support the formation of human vascular networks. This study showed how vascular progenitor cells, including endothelial and mesenchymal stem cells, were encapsulated within a photo-crosslinked porcine-derived GelMA matrix, resulting in the rapid formation of robust vascular networks under pro-angiogenic in vitro conditions. Endothelial cells were responsible for the formation of lumenized capillary-like structures, whereas mesenchymal cells contributed to network stabilization and vessel maturation. Moreover, relatively soft GelMA hydrogels were found to provide the most suitable microenvironment, as they facilitate cell migration and matrix remodeling, two processes that are fundamental for vascular morphogenesis [56].

Another application of porcine-derived GelMA in vascular TE is reported in the study by Parkhideh S. et al., in which GelMA was combined with PEG to fabricate bioprinted matrices with perfusable channels designed to guide vascular organization. After seeding endothelial cells within the channels and incorporating stromal cells into the surrounding matrix, the constructs promoted endothelial organization and vascular maturation. Notably, mature vessels larger than 100 μm formed within the patterned channels, demonstrating that bioprinted GelMA-PEG architectures can direct vascularization and support the development of functional vascularized tissues [80].

GelMA hydrogels have been also used to investigate how matrix stiffness regulates lymphatic endothelial cell behavior and lymphangiogenesis. The regeneration of lymphatic vessels plays a key role in tissue repair processes, contributing to inflammation resolution and the maintenance of tissue homeostasis. In the study conducted by Qin Z. et al., GelMA hydrogels were prepared at three polymer concentrations (5, 7.5, and 15% (*w/v*)), generating matrices with different mechanical behaviors, classified as soft, intermediate, and stiff. Among these formulations, the intermediate-stiffness hydrogel provided the most suitable mechanical environment for the formation of stable lymphatic capillary-like networks. Moreover, the incorporation of vascular endothelial growth factor C, further enhanced this process, suggesting a synergistic effect between mechanical and biochemical cues [81].

The improvement of vascularization, however, does not always require the external incorporation of pro-angiogenic factors. Fish-derived gelatin/GelMA may retain source-dependent biological characteristics that could contribute to the regenerative properties of the resulting hydrogel, although the extent to which specific bioactive components are preserved after gelatin extraction and methacrylation remains formulation dependent [17].

The comparison among GelMA-based hydrogels for vascular and lymphatic TE highlights the need to balance matrix remodelability, structural organization, and biochemical stimulation. Soft GelMA matrices have been shown to promote endothelial migration, lumen formation, and spontaneous vascular network assembly by providing a permissive environment for cell-driven matrix remodeling [82]. However, these formulations may provide limited control over vessel geometry and may not ensure the formation of larger, perfusable vascular structures.

A different strategy relies on patterned or bioprinted GelMA-based constructs, in which predefined channels provide spatial guidance for endothelial organization and vessel maturation. Parkhideh et al. demonstrated that the combination of GelMA with PEG-based components improved the structural stability of bioprinted channels and supported the formation of larger vascular structures within the patterned regions [80]. Nevertheless, the increased structural control may reduce the capacity of cells to remodel the surrounding matrix if the resulting network becomes excessively dense or mechanically restrictive.

The formation of vascular and lymphatic networks is also strongly influenced by the interaction between mechanical and biochemical cues. Qin et al. showed that intermediate GelMA stiffness was more favorable for lymphatic network formation than either softer or stiffer conditions, indicating that maximal matrix compliance is not universally optimal [81]. The additional incorporation of VEGF-C further enhanced lymphatic network formation, although this strategy introduces the need to control factor concentration, retention, and release kinetics [81].

Overall, soft GelMA matrices are particularly suitable for promoting spontaneous capillary self-assembly [82], whereas patterned and polymer-supported formulations provide greater architectural control and are better suited to the fabrication of organized vascular structures [80]. Growth-factor-functionalized systems offer an additional level of biological regulation but require more complex formulation and delivery strategies [81]. Therefore, the optimal GelMA-based hydrogel depends on whether cellular self-organization, geometrical control, or biochemical stimulation represents the main objective of the vascular construct.

### 4.2. Advanced Manufacturing Technologies for GelMA-Based Constructs

Beyond conventional extrusion-based bioprinting, the photo-crosslinkable nature of GelMA enables its processing using advanced manufacturing technologies that provide different advantages in terms of spatial resolution, fabrication speed, and architectural complexity [83]. Among these, digital light processing (DLP), stereolithography (SLA), two-photon polymerization (TPP), volumetric bioprinting (VBP), microfluidic fabrication, and hybrid approaches combining GelMA with electrospun fibers have received increasing attention. However, their suitability depends strongly on the intended application, as each technique operates at different spatial and temporal scales and places distinct demands on GelMA formulation, photochemistry, viscosity, and crosslinking kinetics [83]. DLP enables rapid layer-wise fabrication by projecting entire cross-sectional patterns, allowing cell-laden GelMA constructs to be produced with high geometrical fidelity. Chand et al. [84], for instance, fabricated a GelMA–oxidized carboxymethylcellulose corneal construct with a compressive modulus of 106.3 ± 7.7 kPa, optical transmittance above 90%, and cell viability exceeding 93%. These properties are particularly relevant for corneal applications, where mechanical integrity must be balanced with optical transparency and cytocompatibility. SLA also offers high geometrical accuracy but relies on laser-based photopolymerization. Using this approach, GelMA/hydroxyapatite scaffolds with gradient pore sizes of approximately 50–260 μm have been fabricated [85], illustrating how control over scaffold architecture can be exploited in the design of structurally heterogeneous bone constructs.

Whereas DLP and SLA are suitable for constructing larger three-dimensional architectures, TPP is particularly attractive when microscale or submicroscale control is required. By exploiting localized two-photon polymerization, this technique can generate highly defined structures that would be difficult to achieve using conventional photofabrication methods. Fu and Yu developed a GelMA-based photoresist with a fabrication resolution below 120 nm, extending the use of GelMA to high-precision micro- and nanofabrication [86]. At a larger biological scale, Prina et al. used TPP-fabricated GelMA microstructures to reproduce geometrical features of the human limbal epithelial stem cell niche and showed that these structures could influence the spatial organization and phenotype of human limbal epithelial stem cells [87]. The advantages of TPP in terms of resolution, however, are accompanied by important limitations, particularly the need for specialized equipment and the relatively small fabrication volumes, which currently constrain its scalability.

Other emerging technologies address different limitations of conventional GelMA fabrication. VBP offers the possibility of rapidly generating complex three-dimensional structures without relying on conventional layer-by-layer deposition, while microfluidic approaches provide precise control over microscale geometries and material distribution. Hybrid electrospinning–GelMA strategies offer a different advantage by introducing fibrous reinforcement into the hydrogel network, potentially providing structural features that more closely resemble the fibrous organization of native extracellular matrices. Rather than representing interchangeable alternatives, these fabrication strategies therefore provide complementary routes for controlling GelMA architecture across different length scales. Their effective implementation ultimately requires the fabrication method to be considered together with material-specific variables, including polymer concentration, degree of functionalization, photoinitiator system, optical properties, and crosslinking kinetics.

### 4.3. GelMA-Based Hydrogels for Drug Delivery

Drug delivery refers to the set of strategies developed to transport and release therapeutic agents in a controlled manner, with the aim of improving treatment efficacy and reducing the limitations of conventional administration. Among the different delivery platforms, hydrogels have attracted considerable attention owing to their biocompatibility, high water content, and ability to provide tunable release of bioactive molecules [2].

GelMA-based hydrogels represent promising drug delivery systems (DDS), as they combine the biological advantages of gelatin with customizable physicochemical properties, making them suitable for the localized and controlled release of therapeutic compounds [2]. Their release behavior can be modulated by adjusting parameters such as polymer concentration, crosslinking density, network architecture, and overall composition. In general, increasing GelMA concentration or crosslinking density reduces hydrogel mesh size and limits molecular diffusion, thereby prolonging drug release [61]. However, excessively dense networks may also hinder water uptake and enzyme penetration, potentially slowing hydrogel degradation and restricting payload transport. Therefore, the formulation must be tailored to the physicochemical properties of the therapeutic agent, including its molecular size, solubility, stability, and required release profile.

#### 4.3.1. Anticancer Drug Delivery

DDS can be employed for the release of therapeutic agents of different nature, including anticancer compounds. For example, in the study conducted by Vigata M. et al., a porcine-derived GelMA-based DDS was developed for the sustained local administration of paclitaxel-based Abraxane^®^ in the prevention of breast cancer recurrence after mastectomy. The system showed high drug encapsulation efficiency while preserving the mechanical properties of the hydrogel after loading. Moreover, by varying GelMA concentration, it was possible to modulate mesh size and release kinetics, thereby achieving prolonged drug release over time. Overall, this platform demonstrated effective anticancer activity in vitro, supporting the potential of GelMA hydrogels as a simple and tunable systems for local and sustained drug delivery [88].

This approach illustrates the main advantages of direct drug incorporation into GelMA: limited formulation complexity and the possibility of regulating release through hydrogel concentration and crosslinking conditions. However, release remains strongly dependent on diffusion through the polymer network and on GelMA degradation. The same strategy may therefore be less suitable for therapeutics that are unstable in aqueous environments or require protection from enzymatic degradation.

#### 4.3.2. Antibiotic Delivery

The same research group also designed a GelMA-based DDS for the local release of antibiotics, specifically cefazolin, aimed at the treatment of surgical site infections. Type A porcine GelMA hydrogels with varying polymer concentrations were used to encapsulate different antibiotic doses, achieving very high encapsulation efficiencies without affecting the mechanical properties of the biomaterial. The release profile was influenced by both GelMA concentration and drug loading, showing an initial burst release followed by a diffusion- and degradation-driven release under enzymatic conditions. Moreover, the released cefazolin demonstrated dose-dependent antibacterial activity against S. aureus, confirming the effectiveness of the system [89].

Although the Abraxane^®^- and cefazolin-loaded systems share the same general strategy of direct encapsulation, their release profiles cannot be interpreted independently of drug properties. Molecular size, solubility, interactions with the GelMA network, and loading concentration all contribute to the observed kinetics [89,90]. In antibiotic delivery, an initial burst may be advantageous for rapidly reaching an effective antimicrobial concentration, whereas prolonged anticancer treatment may require a more sustained and uniform release. Thus, the optimal GelMA network density depends not only on the desired release duration but also on the therapeutic function of the delivered compound [89,90].

#### 4.3.3. Growth Factor and Bioactive Molecule Delivery

GelMA-based hydrogels can also be employed as DDS in advanced configurations, such as nanocomposite hydrogels. Direct encapsulation in GelMA may not provide sufficient protection against degradation or rapid diffusion for labile proteins and bioactive molecules. Hierarchical systems combining GelMA with secondary carriers have therefore been developed to provide an additional level of control.

In the study of Yu. et al., a GelMA-based nanocomposite system was developed for the controlled delivery of the chemokine stromal cell-derived factor-1α (SDF-1α) to enhance wound healing. In this approach, SDF-1α was encapsulated within liposomes and embedded into the Type B bovine GelMA matrix to protect the protein from degradation and enable sustained release. The system was shown to effectively promote mesenchymal stem cell migration and activate intracellular signaling pathways associated with tissue regeneration [91].

Another example of GelMA-based hydrogels employed as DDS, is the platform showed by Chen et al. for osteoarthritis treatment through the intra-articular release of sinomenium (SIN). In this case, SIN was encapsulated in chitosan microspheres and embedded within a photo-crosslinked porcine-derived GelMA hydrogel, which served as a supportive matrix for localized and sustained delivery. This combined system demonstrated to reduce cartilage matrix degradation by promoting chondrocyte autophagy and to retard osteoarthritis progression in vivo [82].

Compared with direct drug incorporation, liposome- or microsphere-containing GelMA systems provide greater protection of the therapeutic payload and enable sequential control over release through both the secondary carrier and the surrounding hydrogel [82,91]. This increased control is particularly valuable for unstable proteins or agents requiring prolonged local exposure [91]. However, multicomponent systems are more complex to manufacture, and their overall release behavior depends on interactions among carrier degradation, drug diffusion, GelMA network density, and hydrogel degradation [82,91]. Their advantages should therefore be balanced against greater formulation complexity and the need for reproducible carrier distribution within the hydrogel.

#### 4.3.4. Stimuli-Responsive GelMA Systems

A fish-derived GelMA-based DDS approach instead, is described by the study of Kang et al., in which the material was engineered in the form of nanogels. These fish GelMA nanogels exhibited favorable physicochemical properties, including nanoscale dimensions, low dispersity, and high drug loading efficiency. Moreover, doxorubicin-loaded nanogels showed pH-responsive release behavior and effective inhibition of cell growth, whereas unloaded nanogels maintained high cell viability, highlighting the potential of fish-derived GelMA as a biocompatible carrier for small-molecule drug delivery [92].

Overall, the selection of a GelMA-based drug delivery platform should be guided by the properties of the therapeutic agent and the intended clinical function. Direct loading into GelMA-based hydrogel provides a simple and tunable approach for stable drugs whose release can be controlled through mesh size and degradation [89,90]. Secondary carriers, including liposomes and microspheres, are more suitable when payload protection and multistage release are required [82,91]. Stimuli-responsive nanogels offer enhanced responsiveness to local environmental conditions but differ substantially from bulk hydrogels in terms of retention, administration, and release mechanisms [93]. Thus, no single GelMA formulation is universally superior; each system provides a different balance among formulation simplicity, payload protection, release control, environmental responsiveness, and localized therapeutic retention.

### 4.4. GelMA-Based Hydrogels for In Vitro Models

In vitro models play central roles in biomedical research, as they provide controlled and reproducible platforms for investigating cell behavior, tissue development, disease progression, and responses to therapeutic agents. Nevertheless, most drug discoveries and screening studies are still based on cells cultured on conventional 2D plastic substrates, which fail to capture the complexity of the native in vivo microenvironment, particularly in terms of cell–cell communication and cell–matrix interactions. Biomimetic 3D hydrogel-based systems have emerged as a more physiologically relevant and cost-effective alternative, by closely reproducing the physicochemical features of the native ECM and providing a suitable 3D environment for cell growth [94].

GelMA is therefore considered a promising biomaterial for the fabrication of 3D in vitro models. Its suitability derives from the combination of several advantageous features, including high biocompatibility, intrinsic cell-adhesive sequences, enzymatic degradability, and high-water content [2]. In addition, the possibility of modulating its physicochemical and mechanical properties allows the design of matrices with controlled stiffness, architecture, and biological performance [2]. In this way, GelMA provides a supportive 3D microenvironment that can act as a substitute for the native ECM, promoting cell encapsulation, growth, and tissue-like organization [63].

#### 4.4.1. Gut and Intestinal Models

In the study conducted by Roegiers et al., Type B bovine GelMA was experimentally validated as a scaffold material for small intestinal epithelial cells, with particular focus on permeability, mechanical strength, and biocompatibility. The results showed that both high- and low-substitution GelMA formulations ensured adequate permeability to biologically relevant transport markers, supporting its use in gut in vitro modeling. These GelMA hydrogels were also able to sustain the long-term adhesion and viability of enterocyte-like epithelial cells, as well as co-cultures with mucus-producing cells, demonstrating their suitability for reproducing fundamental features of the intestinal microenvironment [95].

To better reproduce the biochemical characteristics of tissue native microenvironment, GelMA is frequently combined with additional biomolecules or matrix components. An example is provided by the work of Vanhove et al., in which Type A porcine-derived GelMA was supplemented with laminin-111 for the culture and expansion of small porcine intestinal organoids. In GelMA-only hydrogels, organoid polarity was reversed independently of matrix stiffness, indicating that mechanical cues alone were not sufficient to reproduce the basal membrane environment. The addition of laminin-111 partially restored the physiological basal-out organization of the organoids, emphasizing the key role of biochemical signaling in regulating tissue architecture and cell polarity [96].

This comparison demonstrates that, in this intestinal model, modulation of GelMA stiffness alone was insufficient to reproduce the basement-membrane-specific biochemical signals required for correct epithelial organization [96]. The incorporation of ECM proteins such as laminin can therefore improve the biological fidelity of the model by providing tissue-relevant adhesion cues that regulate cell polarity, orientation, and tissue architecture [96]. GelMA alone offers a controllable platform for tuning permeability and mechanical properties [95], but the addition of laminin provides the biochemical cues needed to more accurately reproduce epithelial polarity, orientation, and interactions with the basement membrane [96].

#### 4.4.2. Corneal Models

The versatility of GelMA for in vitro modeling is not limited to intestinal systems, but extends to different tissues and organs, including the cornea. In this regard, a different balance of properties, including transparency, dimensional stability, hydration, and maintenance of the keratocyte phenotype. Alves et al. proposed an innovative fish-derived GelMA-based hydrogel, obtained from gelatin extracted from codfish skin, designed to mimic the ECM of the native corneal stroma. The photo-crosslinked hydrogels, loaded with ascorbic acid, showed suitable swelling behavior, dimensional stability, optical transparency, and handling properties. Human keratocytes encapsulated within the matrix remained viable and metabolically active, while ascorbic acid enhanced cellular activity and supported collagen deposition [79].

This formulation highlights the need to combine appropriate physicochemical properties of the GelMA network with tissue-specific biochemical cues. While the hydrogel provides the structural, swelling, and optical characteristics required for corneal modeling, ascorbic acid enhances cellular activity and collagen synthesis, thereby supporting the development of a more representative stromal matrix. However, its inclusion adds an additional bioactive variable that should be carefully considered when attributing observed cellular responses to the GelMA matrix itself.

#### 4.4.3. Skin Models

In in vitro modeling, scaffold geometry represents a key design parameter, as features such as geometrical confinement, micropatterning, pore size, and interconnectivity, which can strongly influence cell organization, migration, differentiation, and nutrient transport.

Scaffold geometry should therefore be considered not only an intrinsic design parameter, but also a feature that can be accurately controlled through advanced fabrication technologies such as 3D bioprinting. 3D bioprinting has emerged as an effective strategy for the fabrication of GelMA-based hydrogels with defined, reproducible, and application-oriented architectures. In this context, of Tanadchangsaeng et al., employed a fish-derived GelMA bioink for the bioprinting of 3D skin substitutes. The bioink was loaded with adipose-derived mesenchymal stromal cells and human platelet lysate, allowing the generation of cell-laden constructs with favorable biocompatibility and sustained cell viability. Beyond supporting cell survival, the printed scaffolds also demonstrated therapeutic potential in vivo, where they promoted wound healing by enhancing collagen deposition and stimulating neovascularization, particularly at the wound site [97].

GelMA-based 3D in vitro models can also be employed as platforms for testing and validating therapeutic strategies. An example is provided by the study conducted by Villata et al., in which a Type B bovine GelMA-based skin model comprising both epidermal and dermal compartments was developed to investigate wound healing, bacterial infection, and antibiotic treatment responses. Human fibroblasts and keratinocytes were encapsulated within the GelMA matrix to recreate the cellular composition of skin tissue. The model demonstrated the ability to reproduce several key features of native skin, including barrier function, ECM remodeling, inflammatory responses, antimicrobial peptide production, and phenotype changes associated with tissue repair. Moreover, the construct was also able to mimic different responses depending on the bacterial strain involved, and proved to be useful for evaluating antibacterial treatments, revealing that bacterial death itself may trigger distinct inflammatory effects depending on the pathogen [98].

The introduction of multiple cell types and distinct epidermal and dermal compartments enables the model to reproduce a broader range of skin-specific responses, including barrier formation, ECM remodeling, inflammation, bacterial infection, and treatment-dependent effects [98]. This increased biological relevance, however, is accompanied by greater experimental complexity, potentially reducing throughput and increasing inter-sample variability. The choice of model should therefore reflect the intended application: simpler bioprinted cell-laden hydrogels provide greater control over cell distribution and scaffold architecture and are well suited to evaluating tissue organization and regenerative performance [97], whereas multicellular, compartmentalized systems are more informative for investigating the mechanisms underlying infection, inflammatory signaling, and therapeutic response [98].

#### 4.4.4. Vascular Models

Vascular in vitro models illustrate the importance of geometrical control in directing cellular organization. A clear example is provided by the study of Nikkhah et al., in which micropatterned Type A porcine-derived GelMA hydrogels were used to guide the 3D organization of endothelial cells and promote the formation of aligned cord-like structures. In this case, the endothelial cell proliferation, alignment, and cord formation were found to be strongly influenced by the geometry of the patterned features, with constructs of 100 μm in height providing then optimal conditions for the generation of stable and well-organized cords [99].

This approach differs from unpatterned GelMA matrices, in which vascular organization depends mainly on spontaneous cell self-assembly and matrix remodeling. Micropatterning provides greater reproducibility and spatial control, making it advantageous for studying the effect of geometry on endothelial morphogenesis [99]. However, predefined patterns may also constrain cell-driven reorganization and cannot fully reproduce the multiscale complexity of native vascular networks. Accordingly, unpatterned soft GelMA matrices are more suitable for investigating spontaneous vasculogenesis, whereas micropatterned systems provide better control when vessel alignment and geometry are the main experimental variables [99].

#### 4.4.5. Tumor Models

In tumor modeling, a representative application of 3D bio-printed GelMA-based hydrogels is reported in the study of Duan J. et al., in which GelMA-PEGDA composite scaffolds were designed to mimic and reproduce the growth microenvironment of human melanoma cells. The resulting constructs showed suitable mechanical properties and cytocompatibility, supporting long-term tumor cell culture and promoting marked cell aggregation within the scaffold. Compared with conventional 2D cultures, melanoma cells grown in the 3D system exhibited enhanced expansion, differentiation, and a more aggressive phenotype. Moreover, drug testing with luteolin revealed a time- and dose-dependent anticancer effect, with cells cultured in the 3D scaffold showing greater drug resistance, confirming the relevance of this model for anticancer screening [100].

Another application of GelMA-based hydrogels in tumor modeling is described in the study conducted by Shah et al., where these hydrogels were used to establish a 3D glioblastoma model. GelMA enabled the reproduction of the hallmarks of the tumor microenvironment. Patient-derived glioma cells cultured within the hydrogel in fact, displayed phenotypes resembling those found in specific tumor niches, especially hypoxic and perivascular regions, which are commonly associated with more aggressive and treatment-resistant behavior. In addition, the model was able to recapitulate tumor–immune interactions, including macrophage recruitment mediated by cytokine secretion [101]. Compared with structurally reinforced systems this approach places greater emphasis on [102] his finding indicates that tumor model performance cannot be attributed to GelMA concentration alone, as network chemistry, cell seeding strategy, and macroscopic architecture act together to regulate cell aggregation and growth [102]. The cellular heterogeneity and microenvironmental interactions rather than on geometric reproducibility [101]. Villata et al., bio-printed human non-small lung cancer cells encapsulated within different formulations of Type B bovine GelMA, generating constructs with three distinct architectures: round, ring, and grid. The study aimed to investigate how scaffold geometry, GelMA concentration and methacrylation, and cell densities, influence spheroid formation and growth. The best results were obtained using a highly methacrylated 7.5% (*w/v*) GelMA bioink with dispersed single cells, combined with a grid-like architecture, highlighting the importance of both material properties and structural design in recreating physiologically relevant tumor models. Within these constructs, cells were able to form spheroids that more closely mimic the in vivo tumor microenvironment compared to conventional culture systems. The value of these GelMA-based hydrogels lies in their ability to reproduce different aspects of tumor biology rather than in offering a single universally superior platform. Composite GelMA-PEGDA matrices emphasize structural stability and experimental reproducibility, supporting standardized long-term culture and drug-response assessment [100]. Models designed to recreate specific tumor niches instead prioritize cellular heterogeneity, hypoxic and perivascular phenotypes, and tumor–immune interactions [101]. A further level of control is provided by architecture-defined GelMA constructs, which allow the effects of scaffold geometry, network properties, and cell distribution on spheroid formation and tumor growth to be investigated systematically [102]. Together, these approaches show how GelMA can be tailored to isolate and examine distinct determinants of tumor behavior, from drug resistance and niche-dependent phenotypes to architecture-driven cell aggregation.

#### 4.4.6. Osteoarthritis and Cartilage Degradation Models

Another relevant example is the work by Hu Q. et al., in which Type B GelMA was combined with alginate to develop a hydrogel-based model of osteoarthritis-related cartilage degradation. The pathological microenvironment was induced by inflammatory cytokines, including cytokines interleukin-1 beta (IL-1β) and tumor necrosis factor alpha (TNF-α), and the model was used as a preclinical platform to assess the efficacy of MMP-13 inhibitors. After treatment, a reduction in type II collagen degradation was observed, demonstrating how this 3D systems can be employed for in vitro drug screening in degenerative joint diseases [93].

In the GelMA–alginate hydrogel, alginate contributes to the formation and physicochemical regulation of the three-dimensional network, whereas GelMA provides intrinsic cell-adhesive and matrix-remodeling motifs. Although unmodified alginate lacks specific integrin-binding sequences, it can still influence chondrocyte behavior indirectly by regulating cell confinement, matrix stiffness, porosity, and molecular transport. The resulting formulation provided a reproducible platform for modeling inflammation-induced cartilage degradation and evaluating MMP-13 inhibitors [93].

Overall, the selection of a GelMA-based in vitro model should be guided by the level of biological and structural complexity required by the experimental question. GelMA-only systems provide comparatively simple and controllable platforms for studying the effects of stiffness, permeability, and cell encapsulation [81,95]. The addition of tissue-specific ECM molecules improves biochemical fidelity and may be essential for reproducing polarity, differentiation, or phenotype maintenance [96]. Synthetic polymers and composite networks provide greater structural stability and reproducibility [100], whereas micropatterning and bioprinting introduce spatial control over cell and tissue organization [97,99,102]. Multicellular and disease-specific constructs can provide greater physiological relevance, although their increased biological complexity may also make standardization and high-throughput implementation more challenging. No single formulation is therefore optimal for all in vitro models; each represents a different compromise among controllability, reproducibility, structural definition, biochemical fidelity, and biological complexity.

## 5. Comparative Overview of GelMA Applications

### Summary Table of GelMA-Based Systems

The representative GelMA-based systems discussed throughout Section 4 are summarized in Table 7.

## 6. Translational Challenges of GelMA-Based Hydrogels

Despite the extensive body of evidence supporting the versatility of GelMA hydrogels in tissue engineering, drug delivery, 3D cell culture, and bioprinting, their transition from laboratory-scale research platforms to clinically relevant products remain challenging. In contrast to proof-of-concept studies, clinical translation requires not only reproducible biological performance but also well-defined raw materials, standardized manufacturing processes, validated sterilization procedures, appropriate quality control, scalable production, regulatory compliance, and convincing evidence of long-term safety. Importantly, these requirements are interconnected: changes introduced during synthesis, purification, sterilization, or scale-up may alter the physicochemical properties of GelMA and consequently affect its biological performance. Therefore, translational development requires a shift from formulation optimization at the laboratory scale toward reproducible, quality-controlled manufacturing processes.

### 6.1. Sterilization and Contamination Control

The biological origin of gelatin is an important consideration in the translational development of gelatin- and GelMA-based biomaterials. Beyond source-dependent characteristics such as gelatin type, extraction process, Bloom strength, and molecular weight distribution, the microbiological quality of the starting material can influence the safety and reproducibility of the resulting hydrogel. Endotoxin contamination is particularly relevant, although it is not routinely reported. Kamiya et al. [103] identified substantial variability in endotoxin content among commercially available gelatin and GelMA preparations, with some materials exceeding 1000 EU/mL. Endotoxin levels were not associated with the degree of methacryloyl functionalization, suggesting that contamination is more closely related to the quality and processing history of the gelatin precursor. Moreover, endotoxin-containing hydrogels altered inflammatory cytokine secretion and chondrogenic differentiation, potentially affecting the interpretation and reproducibility of biological results [103].

Adequate purification is also required to remove unreacted reagents and low-molecular-weight products generated during GelMA synthesis. Dialysis followed by lyophilization is commonly employed [7], but differences in purification conditions may introduce additional variability among GelMA preparations. Purification should therefore be considered part of the definition of reproducible manufacturing specifications, particularly for clinical-grade materials.

Sterilization represents another critical source of variability, as its effects depend on both the method and the stage at which it is applied. Rizwan et al. [104] showed that autoclaving and ethylene oxide (EtO) treatment reduced GelMA stiffness, whereas γ-irradiation increased stiffness, decreased pore size, and slowed degradation. When applied to GelMA powder, γ-irradiation also impaired subsequent hydrogel formation. Interestingly, Zhang et al. [105] reported relatively limited changes in morphology, swelling behavior, elastic modulus, and cell viability following different sterilization treatments, while observing significant alterations in macrophage gene-expression profiles. Together, these findings indicate that conventional physicochemical characterization alone may not capture biologically relevant effects induced by sterilization.

For translational applications, precursor quality, purification, and sterilization should therefore be considered interconnected components of GelMA manufacturing. Sterilization procedures must ensure microbial safety while preserving the physicochemical and biological properties required for the intended application. This is particularly important for implantable or tissue-contacting systems, where sterility, pyrogen control, reproducibility, and material quality are essential requirements.

### 6.2. Reproducibility, Batch-to-Batch Variability, and Standardization

Reproducibility represents an important consideration for the translation of GelMA from a highly tunable laboratory material to a standardized biomaterial platform. However, a distinction should be made between intrinsic limitations of GelMA and variability introduced by the starting material and manufacturing process. Although GelMA synthesis is conceptually straightforward, its final properties depend on multiple variables, including the source and type of gelatin, Bloom strength, molecular weight distribution, methacrylic anhydride-to-gelatin ratio, degree of methacryloyl functionalization, purification procedure, polymer concentration, photoinitiator, wavelength and intensity of light exposure, and crosslinking time. Consequently, insufficient control of these parameters may result in apparently similar GelMA formulations exhibiting different physicochemical and biological properties.

Gelatin source and batch are significant sources of variability. Comparative studies using different commercial gelatin preparations have demonstrated that species, Bloom strength, and batch of the starting gelatin can influence the degree of functionalization obtained during methacrylation. Importantly, differences between batches of the same gelatin source may also affect the properties of the resulting GelMA, including hydrogel elasticity [106]. Such findings highlight the importance of controlling the characteristics of the starting material and defining appropriate acceptance criteria before synthesis.

Nevertheless, this variability is not necessarily inherent to GelMA. Zhu et al. [8] demonstrated that a controlled one-pot synthesis strategy could produce GelMA batches with high consistency in degree of substitution, secondary structure, swelling, stiffness, degradation, and cell viability across five independent batches. These results show that variability can be substantially reduced through appropriate control of the synthesis process and that reproducible GelMA manufacturing is achievable when critical processing parameters are adequately defined [8]. Thus, the translational challenge is not simply to eliminate variability but to identify, control, and standardize the parameters responsible for it.

A further challenge is the lack of universally adopted standards for GelMA characterization and reporting. The degree of substitution or functionalization is a major determinant of crosslinking density and, consequently, swelling, degradation, mechanical properties, and biological behavior. However, differences in terminology, analytical methods, and experimental conditions complicate direct comparisons between studies. He et al. emphasized the need for greater standardization of GelMA characterization and proposed measurable parameters to establish more consistent relationships between material structure and performance [14].

For translational development, standardization should extend beyond the degree of functionalization to include gelatin source and specifications, molecular weight distribution, Bloom strength, residual reagents and endotoxin levels, GelMA concentration, photoinitiator system, crosslinking conditions, rheological and mechanical properties, degradation kinetics, sterilization, and storage conditions. Defining relevant parameters as critical quality attributes could improve reproducibility across batches, laboratories, and manufacturing scales while providing a stronger basis for quality-by-design approaches.

### 6.3. Scalability, Manufacturing, and Cost

Another challenge for the clinical translation of GelMA is the transition from laboratory-scale synthesis to scalable and reproducible manufacturing. Most GelMA formulations described in the literature are prepared in small batches, with several steps, including synthesis, purification, lyophilization, solution preparation, and photopolymerization, performed under laboratory conditions. Although suitable for experimental studies, these procedures may be difficult to transfer directly to clinical manufacturing.

Increasing the production volume can affect reaction kinetics, mixing, heat and mass transfer, purification, and drying. Dialysis, for example, is widely used for GelMA purification at laboratory scale but may become time-consuming and difficult to control as production volumes increase. Sterilization and aseptic processing add further complexity, as they must ensure product safety without altering material properties. Similar difficulties are encountered with other hydrogel-based biomaterials, for which laboratory protocols often require considerable optimization before they can be transferred to larger-scale production [50].

For GelMA, scale-up is also closely related to the variability of the gelatin precursor. Differences in gelatin source and batch can affect methacryloyl functionalization and the properties of the resulting hydrogel. More consistent production would therefore require defined specifications for the starting gelatin, together with appropriate controls of relevant material attributes. These may include the degree of functionalization, molecular weight distribution, endotoxin content, residual reagents, rheological properties, and crosslinking behavior.

The economic feasibility of GelMA should also be evaluated beyond the cost of starting reagents. Gelatin and methacrylic anhydride are relatively accessible, making laboratory-scale GelMA production comparatively inexpensive. Clinical manufacturing, however, would also involve qualified raw materials, controlled production environments, purification and sterile processing, analytical testing, validated storage and packaging, and batch release. The overall cost of a clinically compliant GelMA product may therefore differ substantially from that estimated from reagent costs alone.

Moving toward larger-scale production will require manufacturing considerations to be addressed alongside formulation development. Automation and more controlled mixing, purification, and sterilization procedures could reduce operator-dependent variability and facilitate scale-up. This will be particularly relevant when large quantities of GelMA or highly consistent patient-specific products are required.

### 6.4. Regulatory Considerations: GMP, FDA, and EMA

The clinical translation of GelMA-based hydrogels also depends on the regulatory requirements governing manufacturing, quality control, sterility, and biological safety. GelMA does not fall under a single regulatory classification, as the applicable pathway depends on the intended use, composition, mode of action, and final product. For example, a GelMA-based material may be developed as a scaffold, drug-delivery matrix, medical-device component, or part of a combination product, with different regulatory requirements applying in each case.

In the United States, when a GelMA-based product falls within the medical-device framework, manufacturing is subject to the FDA Quality Management System Regulation (QMSR), which became effective on 2 February 2026, and incorporates ISO 13485:2016 by reference (FDA, 2026). For sterile devices, FDA guidance additionally recommends appropriate information concerning the sterilization process and its validation, together with relevant pyrogenicity information, in premarket submissions (FDA, 2024). Endotoxin and pyrogen control are also addressed in current FDA guidance on pyrogen and endotoxin testing, which covers devices, drugs, and biological products (FDA, 2026). Furthermore, FDA guidance implementing ISO 10993-1 emphasizes a risk-based approach to biological evaluation, including consideration of chemical characterization and potential biological responses to device materials (FDA, 2023).

Within the European Union, medicinal products are manufactured according to the EU Good Manufacturing Practice framework established in EudraLex Volume 4, including requirements relevant to sterile manufacturing (European Commission, 2024). The EMA guideline on sterilization provides recommendations for the selection and documentation of sterilization approaches for medicinal products, active substances, excipients, and primary containers (EMA, 2019). When a GelMA-based system incorporates a medicinal product and a medical-device component, additional requirements may apply under the regulatory framework for drug–device combinations. In particular, EMA guidance addresses quality documentation for medicinal products used with medical devices and should be considered together with the requirements of the Medical Devices Regulation, including Article 117 (EMA, 2021/2022).

Although a detailed discussion of individual regulatory pathways is beyond the scope of this review, these frameworks illustrate several issues that need to be addressed during GelMA product development, including controlled manufacturing, validated sterilization, endotoxin and chemical control, traceability, and biological safety. Addressing these requirements early in development may facilitate the transition from experimental GelMA formulations to clinically applicable products.

### 6.5. Long-Term Biosafety and Clinical Translation

Successful clinical translation requires evidence of long-term safety, stability, and functional performance, aspects that remain less extensively investigated than short-term cytocompatibility for many GelMA-based systems. GelMA is attractive for biomedical applications because its gelatin-derived composition provides cell-adhesive motifs and enzymatically degradable sequences. However, these same features may result in relatively rapid degradation under certain physiological conditions. Degradation kinetics must therefore be matched to the rate of tissue regeneration and the mechanical requirements of the intended application.

Long-term safety assessments should also account for degradation products, residual reagents, photoinitiators, sterilization-induced modifications, and potential immune responses. Despite the general biocompatibility of gelatin, its biological origin raises additional concerns related to source, purification, endotoxin burden, and compositional variability. Sterilization adds another layer of complexity, as it can affect both material properties and biological responses. For example, Zhang et al. observed changes in macrophage gene-expression profiles following different sterilization treatments [105].

A further limitation is that evidence for many GelMA applications remains predominantly preclinical, with most studies relying on in vitro models and small animals. While these studies provide valuable information on feasibility and biological activity, they cannot fully predict long-term behavior in humans. Larger animal models and, ultimately, appropriately designed clinical studies will be needed, particularly for applications in which degradation, mechanical stability, tissue integration, or immune responses are critical to functional outcomes.

Clinical translation of GelMA therefore requires more than evidence of short-term biocompatibility or efficacy. Predictable and reproducible performance must be demonstrated throughout the product lifecycle, linking material characterization and manufacturing control with sterilization, long-term safety assessment, appropriate preclinical models, and regulatory requirements. Integrating these aspects early in development will be important for moving GelMA from an experimental biomaterial toward a clinically viable platform.

## 7. Current Challenges and Future Perspectives

Despite the extensive evidence supporting the versatility of GelMA-based hydrogels, several challenges continue to limit the reproducibility, comparability, and broader translation of these systems. The future development of GelMA should move beyond generating new formulations or expanding its range of applications, but rather on a clearer understanding of the relationships between material characteristics, fabrication processes, and biological outcomes. Key priorities include the standardization of GelMA platforms, the development of multifunctional and biomimetic systems, the integration of advanced biofabrication with patient-specific models, and the translating laboratory-scale proof-of-concept studies into reproducible and clinically relevant products [14].

### 7.1. Standardization and Reproducibility

A key priority for future GelMA research is to improve consistency in material characterization and reporting. As discussed throughout this review, gelatin source, Type A or Type B classification, Bloom strength, molecular weight distribution, degree of functionalization, polymer concentration, purification, and crosslinking conditions can all influence hydrogel behavior [24,25]. Rather than defining a single “optimal” GelMA formulation, efforts should focus on establishing material specifications and reporting criteria that allow meaningful comparisons across formulations and laboratories.

This requires detailed reporting of both precursor characteristics and processing parameters, as well as harmonized methods for assessing the degree of functionalization and relevant physicochemical, mechanical, and biological properties. Greater consistency in these areas would help clarify structure–property–function relationships and make it easier to determine whether differences between studies reflect the GelMA formulation itself or variations in preparation and characterization methods.

Improved standardization will be particularly important for applications that depend on high reproducibility, such as patient-derived models, high-throughput screening, and clinical manufacturing.

### 7.2. From Tunable Hydrogels to Multifunctional and Biomimetic Platforms

The next generation of GelMA systems is also expected to move beyond the concept of a hydrogel whose properties are primarily tuned through polymer concentration and degree of functionalization. Although these parameters provide substantial control over network mechanics, swelling, degradation, and transport, increasingly biomimetic applications will require the simultaneous integration of mechanical, biochemical, and dynamic cues.

Future research should therefore focus on multifunctional GelMA-based systems in which additional extracellular matrix components, peptides, synthetic polymers, nanoparticles, or bioactive molecules are incorporated according to the requirements of the target tissue or disease model [62,107]. The aim, however, should not be to increase material complexity for its own sake, but to design systems in which each component has a defined biological or physicochemical role.

This approach could be particularly valuable for tissues in which GelMA alone cannot reproduce the complexity of the native extracellular environment. For example, the incorporation of laminin into intestinal GelMA models demonstrated that biochemical cues could complement the mechanical properties of the hydrogel and improve the reproduction of epithelial polarity and tissue organization [3].

The remaining challenge is to determine which combinations of biochemical and biophysical cues are necessary to achieve physiologically relevant microenvironments without introducing unnecessary complexity or compromising reproducibility and manufacturability.

### 7.3. Advanced Biofabrication and Personalized Models

Advanced biofabrication offers a way to translate the increasing complexity of GelMA-based materials into spatially organized biological environments. Beyond improving printing resolution, these technologies can be used to control the distribution of material compositions, cell populations, and biochemical cues within a single construct. Combining GelMA with extrusion-based bioprinting, stereolithography, volumetric bioprinting, and other emerging approaches may therefore support the fabrication of more tissue-specific architectures [3,58].

A particularly promising direction is the integration of GelMA matrices with patient-derived organoids and other advanced three-dimensional models. Here, GelMA tunability can be exploited not only to support cell growth but also to reproduce selected features of tissue- or disease-specific microenvironments. This is especially relevant to cancer models, where matrix properties and tumor–microenvironment interactions can influence cellular phenotype and therapeutic response [100,101].

Combining patient-derived cells or organoids with compositionally and mechanically defined GelMA matrices may improve the physiological relevance of drug-screening platforms and support their use in personalized medicine. Whether these systems can reliably retain patient-specific molecular and functional characteristics over prolonged culture, however, remains to be established. Addressing this question will require validation across multiple patient samples and experimental conditions.

### 7.4. From Proof-of-Concept to Clinical Translation

A remaining challenge is to translate the versatility of GelMA demonstrated at laboratory scale into reproducible, scalable, and clinically relevant products. As discussed in Section 6, improving biological performance alone will not be sufficient. Material standardization, manufacturing, quality control, sterilization, safety assessment, and regulatory requirements need to be considered early in product development.

Scale-up must also preserve the material attributes established during laboratory development, including precursor quality, degree of functionalization, molecular characteristics, network properties, and biological performance. This is particularly relevant to patient-specific applications, where customization must be balanced against manufacturing reproducibility and quality assurance.

Linking formulation and processing parameters to predefined quality attributes could provide a more systematic basis for controlling batch-to-batch variability and supporting regulatory assessment. Clinical translation will ultimately depend on whether biological efficacy can be combined with consistent manufacturing, long-term safety, scalability, and a regulatory pathway appropriate to the intended application.

Moving GelMA from a versatile research material toward clinical use will therefore require more than continued formulation optimization. The emphasis should increasingly shift from proof-of-concept development to application-specific materials designed with reproducibility, manufacturing, and quality control in mind.

## 8. Conclusions

This review provides an integrated perspective on the transition from gelatin to GelMA, examining how the source, composition, and physicochemical characteristics of the gelatin precursor influence methacryloyl functionalization, hydrogel network formation, and ultimately material performance. Particular attention has been paid to Type A and Type B gelatin and to the combined effects of degree of functionalization, polymer concentration, crosslinking conditions, and processing parameters. From this source-to-performance perspective, GelMA cannot be considered a single standardized material. Its properties reflect both the characteristics of the gelatin precursor and the conditions used during synthesis and processing [24,25]. Formulations described simply as “GelMA”, or even prepared at the same nominal polymer concentration, may therefore differ substantially in their final properties.

The versatility of GelMA arises from its combination of biocompatibility, bioactivity, accessibility, and tunable physicochemical and mechanical properties. At the same time, this tunability makes careful consideration of the gelatin precursor and processing history essential. Understanding differences in gelatin source, extraction process, composition, Bloom strength, and molecular characteristics can help explain variations in GelMA behavior and support a more rational selection of materials for specific applications. Rather than relying primarily on empirical optimization, future development should aim to establish clearer structure–property–function relationships.

Achieving greater reproducibility will require attention to the entire material-development chain, from gelatin source and molecular characteristics to methacryloyl functionalization, purification, formulation, and photopolymerization. Variability introduced at any of these stages may influence network formation and, in turn, the mechanical and rheological properties, swelling behavior, porosity, degradation, and biological performance of the final hydrogel [14,23,24]. A better understanding and control of these relationships will be central to the development of reproducible, application-specific GelMA systems.

Advanced biofabrication and increasingly sophisticated three-dimensional models may further extend the applications of GelMA. Combining controlled GelMA matrices with emerging fabrication technologies and patient-derived models offers opportunities to create more physiologically relevant microenvironments for disease modeling and therapeutic screening [3]. This is particularly relevant to patient-derived organoids, where matrix properties can be adjusted to reproduce selected features of tissue- and disease-specific environments. GelMA-based tumor models have already reproduced aspects of tumor behavior, including aggressive phenotypes, tumor–microenvironment interactions, and altered therapeutic responses [100,101]. Integrating such models with high-throughput drug screening may eventually provide more predictive platforms for personalized medicine.

Translation beyond the laboratory, however, remains challenging. Variability in gelatin source, functionalization, purification, crosslinking, and sterilization can affect the reproducibility of GelMA and complicate comparisons between studies. Progress in this area will require greater consistency in material specifications, manufacturing, characterization, and reporting rather than the adoption of a single universal formulation. Relevant information should include the characteristics of the gelatin precursor, degree of functionalization and its analytical determination, purification and formulation conditions, and photopolymerization parameters. Consistent characterization of mechanical and rheological properties, swelling, degradation, porosity, and biological performance would further improve cross-study comparability [14,24]. These considerations become even more important for clinical translation, where reproducibility must be accompanied by scalable manufacturing, safety, and quality assurance.

GelMA is therefore better viewed as a modular biomaterial platform than as a single, uniformly defined hydrogel. Its performance emerges from the combined effects of the gelatin precursor, functionalization, formulation, and processing, while its versatility allows these variables to be adapted to different biological requirements. Standardization should not aim to eliminate this flexibility, but to provide sufficient material definition and reporting to make different GelMA systems reproducible and meaningfully comparable. Together with advances in biofabrication, biomimetic design, and patient-derived models, greater control over these variables may support the development of GelMA from a widely used experimental material into a more reproducible and application specific biomedical platform.

## Figures and Tables

**Table 1:** Comparison between gelatin and GelMA.

Property	Gelatin	GelMA
Origin	Collagen hydrolysis	Methacrylated gelatin
Crosslinking	Physical, chemical, enzymatic	Mainly photo-crosslinking
Thermoresponsive	Yes	Reduced
Mechanical stability	Low	Tunable, higher
Cell adhesion	Excellent (RGD)	Excellent (RGD retained)
Degradation	Fast	Tunable
Printability	Limited	Excellent
Main applications	Drug carriers, simple hydrogels	TE, DDS, bioprinting, in vitro models

**Table 2:** Summary of the main differences among commonly used GelMA photoinitiating systems in terms of activation conditions, curing efficiency, cytocompatibility, and practical limitations.

Photoinitiator	Activation	Curing and Cytocompatibility	Main Considerations	Ref.
I2959	Type I; UV, ~365 nm	Good viability at 0.3–0.5% (*w/v*); lower than LAP at 0.7–0.9%	Well established; limited curing efficiency and UV penetration	Xu et al. [52]
LAP	Type I; UV/violet, ~365–405 nm	Rapid gelation (≤12 s at 5% GelMA; ~6 s at 10%); good cytocompatibility at 0.01–0.5% (*w/v*)	Fast, simple system; toxicity depends on concentration and light dose	[11,14]
EY	Type II; green light, ~500–550 nm	Gelation ~40 s; highest viability at 0.01 mM, decreasing above 0.02 mM	Visible-light activation; requires co-initiator/co-monomer	[12,14]
Ru/SPS	Type II; blue light, ~400–450 nm	Gelation ~5–10 s; high viability at 0.05–0.3 mM Ru	Rapid curing and high penetration (up to 10 mm); SPS-dependent toxicity	[13,14]

**Table 3:** Principal formulation and processing parameters controlling GelMA-based hydrogels mechanical and rheological properties, together with representative quantitative values and their main effects on network behavior.

Factor	Effect on Mechanical/Rheological Properties	Representative Findings	Ref.
GelMA concentration	↑ stiffness and Young’s modulus; generally, ↑ G′ and G″	Young’s modulus: 1.55 kPa at 5% → 48.50 kPa at 15% (*w/v*), due to increased polymer density and crosslinking.	[53,55]
DS	↑ compressive modulus and matrix rigidity	Compressive modulus: 2.0 → 4.5 kPa as DS increases from 49.8% to 73.2%, reflecting higher crosslink density.	[7,56]
Bloom number	Higher Bloom generally associated with greater stiffness and viscoelasticity	Lower Bloom values are associated with reduced stiffness and viscoelasticity.	[24]
Photoinitiator concentration/type	Modulates crosslink density and final stiffness	Higher photoinitiator concentration generally enhances crosslinking, while LAP remains effective at relatively low concentrations.	[12,14,20,25]
Light exposure time	↑ mechanical properties until crosslinking reaches a plateau	Tensile properties increase with exposure time until reaching a plateau after crosslinking.	[12,13]
Rheological response	G′ and G″ generally ↑ with GelMA concentration	G′ represents the elastic component and G″ the viscous component. Their crossover can be used to identify the sol–gel transition.	[14]
Temperature/GelMA origin/crosslinking conditions	Influence gelation kinetics and viscoelastic response	Affect the evolution of G′ and G″ and therefore the position of the sol–gel transition.	[36,58]

↑ = increment.

**Table 4:** Main mechanical testing methods used for GelMA-based hydrogels, parameters measured, and principal factors affecting the reported values.

Testing Method	Main Parameters	Mechanical Information	Main Factors Affecting Values
Compression	Compressive modulus	Bulk response to compressive loading	Strain range, loading rate, geometry, hydration
Tensile testing	Young’s modulus, tensile strength, strain at failure	Bulk response to elongation	Strain rate, geometry, sample defects
Oscillatory rheology	G′, G″	Viscoelastic response under shear	Strain, frequency, temperature, geometry
Oscillatory DMA	E′, E″	Time-dependent viscoelastic response under axial cyclic loading	Frequency, strain amplitude, loading mode
Stress relaxation (DMA)	Stress relaxation, residual stress	Time-dependent stress dissipation at constant strain	Applied strain, relaxation time, loading protocol
AFM nanoindentation	Local/indentation modulus	Local nanoscale stiffness and heterogeneity	Probe geometry, indentation rate/depth, contact model

**Table 5:** Main formulation and processing parameters governing GelMA properties.

Parameter	Increase Leads to	Main Effect on Hydrogel
GelMA concentration	↑ polymer content	↑ crosslink density, ↑ stiffness, ↓ swelling, ↓ pore size
DS	↑ methacrylation	↑ elastic modulus, ↓ swelling, ↓ degradation
DoF	↑ crosslinking	↑ rigidity, ↓ water uptake
UV light intensity	↑ radical generation rate	↑ polymerization rate, ↓ pore size, potential ↓ cell viability
UV exposure time	↑ polymerization	↑ stiffness (until plateau)
Photoinitiator concentration	↑ radicals	↑ crosslinking (within optimal range)
Bloom number	↑ triple helices	↑ gel strength and mechanical performance

↑ = Increase; ↓ = Decrease.

**Table 7:** Comparative applications summary of representative GelMA-based hydrogels systems and their biomedical applications.

Author	Publication Year	Type of GelMA	Application
**Zhao X. et al.** [65]	2016	Porcine GelMA	Skin TE; formation of a multilayered epidermis with functional barrier properties.
**Jahan I. et al.** [66]	2019	Bovine GelMA	Wound healing; enhanced wound closure and antibacterial activity through incorporation of silver nanoparticles.
**Visser J. et al.** [71]	2015	Type A porcine GelMA	Cartilage repair; mechanical reinforcement of GelMA hydrogels through combination with PCL.
**Chen J. et al.** [72]	2020	Type B bovine GelMA	Cartilage TE; guidance of chondrogenic differentiation of bone marrow-derived mesenchymal stem cells and maintenance of the cartilaginous phenotype.
**Machado I. et al.** [75]	2023	Fish GelMA	Cartilage TE; development of photo-crosslinked hybrid hydrogels with methacrylated hyaluronic acid and chondroitin sulphate to improve chondrocyte viability.
**Wang Y. et al.** [76]	2018	Bovine GelMA	Bone TE; development of GelMA/PEGDA hybrid hydrogels with improved mechanical stability, controlled degradation, and osteoblast adhesion and proliferation.
**Zhao X. et al.** [77]	2016	Porcine GelMA	Stem cell-based bone TE; microsphere system for encapsulation of bone marrow-derived mesenchymal stem cells and growth factors, supporting osteogenic differentiation and bone formation.
**Huang L. et al.** [78]	2020	Fish GelMA	Bone TE; development of biomimetic hydrogels incorporating nano-sized fish bone to improve mechanical performance, modulate the immune microenvironment, and promote bone regeneration.
**Chen Y. et al.** [56]	2012	Porcine GelMA	Vascular TE; formation of human vascular networks from encapsulated endothelial and mesenchymal stem cells within photo-crosslinked GelMA hydrogels.
**Parkhideh S. et al.** [80]	2022	Porcine GelMA	Vascular TE; fabrication of bioprinted PEG/GelMA matrices with perfusable channels to guide endothelial organization, vascular maturation, and vascularized tissue formation.
**Qin Z. et al.** [81]	2024	GelMA	Lymphatic TE; investigation of matrix stiffness and VEGF-C cues in lymphatic capillary-like network formation.
**Vigata M. et al.** [88]	2020	Porcine GelMA	Drug delivery; sustained local release of paclitaxel-based Abraxane^®^ for prevention of breast cancer recurrence after mastectomy.
**Vigata M. et al.** [89]	2021	Type A porcine GelMA	Drug delivery; local release of cefazolin for treatment of surgical site infections, with dose-dependent antibacterial activity against *S. aureus*.
**Yu J. R. et al.** [91]	2020	Type B bovine GelMA	Drug delivery; nanocomposite hydrogel for sustained SDF-1α release to enhance wound healing and promote mesenchymal stem cell migration.
**Chen P. et al.** [82]	2016	Porcine GelMA	Drug delivery; intra-articular sustained release of sinomenium for osteoarthritis treatment, reducing cartilage matrix degradation and delaying disease progression.
**Kang M. G. et al.** [92]	2019	Fish GelMA	Drug delivery; pH-responsive fish GelMA nanogels for small-molecule delivery, with doxorubicin loading and effective inhibition of cell growth.
**Roegiers I. et al.** [95]	2025	Type B bovine GelMA	In vitro gut modeling; scaffold for small intestinal epithelial cells, supporting permeability, biocompatibility, and long-term epithelial cell adhesion and viability.
**Vanhove L. et al.** [96]	2025	Type A porcine GelMA	In vitro intestinal organoid modeling; laminin-111-supplemented hydrogel for small intestinal organoid culture, supporting basal-out organization and physiological cell polarity.
**Alves A. et al.** [79]	2023	Fish GelMA	In vitro corneal modeling; hydrogel mimicking the native corneal stroma and supporting keratocyte viability, metabolic activity, and collagen deposition.
**Tanadchangsaeng N. et al.** [97]	2024	Fish GelMA	In vitro skin modeling; 3D bioprinted skin substitutes loaded with adipose-derived mesenchymal stromal cells and human platelet lysate, supporting cell viability and promoting wound healing.
**Villata S. et al.** [98]	2024	Type B bovine GelMA	In vitro skin modeling; 3D skin model for studying wound healing, bacterial infection, and antibiotic treatment responses.
**Nikkhah M. et al.** [99]	2012	Type A porcine GelMA	In vitro vascular modeling; micropatterned hydrogels guiding endothelial cell alignment, proliferation, and formation of organized cord-like structures.
**Duan J. et al.** [100]	2022	GelMA	In vitro tumor modeling; 3D bioprinted GelMA/PEGDA composite scaffolds for melanoma cell culture and anticancer drug screening.
**Shah N. et al.** [101]	2021	GelMA	In vitro tumor modeling; 3D glioblastoma model reproducing tumor-niche phenotypes, including hypoxic and perivascular features, and tumor–immune interactions.
**Villata S. et al.** [102]	2023	Type B bovine GelMA	In vitro tumor modeling; 3D bioprinted GelMA constructs for lung tumor spheroid formation, investigating the effects of GelMA properties, cell density, and scaffold architecture.
**Hu Q. et al.** [93]	2024	Type B GelMA	In vitro osteoarthritis modeling; GelMA–alginate hydrogel for cytokine-induced cartilage degradation and screening of MMP-13 inhibitors.

## Data Availability

No new data were created or analyzed in this study. Data sharing is not applicable to this article.
